# Molluscicidal activity and physiological toxicity of quaternary benzo[c]phenanthridine alkaloids (QBAs) from *Macleaya cordata* fruits on *Oncomelania hupensis*

**DOI:** 10.1371/journal.pntd.0007740

**Published:** 2019-10-11

**Authors:** Wenshan Ke, Chang Tu, Dezhi Cao, Xiong Lin, Qiqiang Sun, Qian Zhang

**Affiliations:** 1 Green Resources Transformation and Collaborative Innovation Center, and State Key Laboratory of Biocatalysis and Enzyme Engineering, School of Life Science, Hubei University, Wuhan, PR China; 2 The First Affiliated Hospital of Huanghuai University, Zhumadian, PR China; 3 Research Institute of Forestry Chinese Academy of Forestry, Beijing, PR China; University of the District of Columbia, George Washington University School of Medicine and Health Sciences, UNITED STATES

## Abstract

Schistosomiasis is a serious worldwide parasitic disease. One of the best ways to control schistosomiasis is to control the population of *Oncomelania hupensis* snails. We sought to identify a high-efficiency biogenic molluscicide against *Oncomelania* with low toxicity, to avoid chemical molluscicide contamination and toxicity in aquatic organisms. We extracted quaternary benzo[c]phenanthridine alkaloids (QBAs) from *Macleaya cordata* fruits. Molluscicidal activity of the QBAs against *Oncomelania* was determined using bioassay. Our results showed that the extracted QBAs had a strong molluscicidal effect. In treatment of *O*. *hupensis* with QBAs for 48 h and 72 h, the lethal concentration (LC_50_) was 2.89 mg/L and 1.29 mg/L, respectively. The molluscicidal activity of QBAs was close to that of niclosamide (ethanolamine salt), indicating that QBAs have potential development value as novel biogenic molluscicides. We also analyzed physiological toxicity mechanisms by examining the activity of several important detoxification enzymes. We measured the effect of the extracted QBAs on the activities of glutathione S-transferase (GST), carboxylesterase (CarE), acid phosphatase (ACP), and alkaline phosphatase (AKP) in the liver of *O*. *hupensis*. We found that the effects of QBAs on detoxification metabolism in *O*. *hupensis* were time and concentration dependent. The activities of GST, CarE, AKP, and ACP in the liver of snails increased significantly in the early stage of treatment (24 h), but decreased sharply in later stages (120 h), compared with these activities in controls. GST, CarE, AKP, and ACP activity in the liver of snails treated with LC_50_ QBAs for 120 h decreased by 62.3%, 78.1%, 59.2%, and 68.6%, respectively. Our results indicate that these enzymes were seriously inhibited by the extracted QBAs and the detoxification and metabolic functions of the liver gradually weakened, leading to poisoning, which could be the main cause of death in *O*. *hupensis* snails.

## Author summary

Quaternary benzo[c]phenanthridine alkaloids (QBAs) have antimicrobial, antifungal, anti-inflammatory, and anticancer activities (anticancer, antibiosic, and antiphlogistic functions) and have been explored for use as bioantibiotics in livestock. *Macleaya cordata* fruits have a high QBA content. We isolated QBAs from *M*. *cordata* to evaluate molluscicidal activity against *Oncomelania* and to analyze the physiological toxicity mechanisms. Our results showed that QBAs had strong molluscicidal activity, similar to that of niclosamide. The activities of several important detoxification enzymes (glutathione S-transferase, carboxylesterase, acid phosphatase, and alkaline phosphatase) in the liver of *O*. *hupensis* snails were severely inhibited by QBAs. The detoxification and metabolic liver functions gradually weakened via poisoning, which could be the main cause of death in these snails. In China, there are abundant wild plant resources of *M*. *cordata*, which is often used in traditional Chinese medicine. QBAs comprising a natural combination of sanguinarine and chelerythrine bisulfate in about a 2:1 ratio had stronger molluscicidal activity than sanguinarine alone. This natural composition could serve as a scientific basis for future development of novel biomolluscicides using these active ingredients and proportions, which could provide an environmentally friendly alternative to the problem of contamination with chemical molluscicides used to combat schistosomiasis in endemic areas.

## Introduction

Schistosomiasis is an acute and chronic parasitic disease that is caused by blood flukes of the genus *Schistosoma*. Almost 240 million people worldwide are at risk of schistosomiasis, and at least 206.4 million people required preventive treatment for the disease in 2016 [1]. *Schistosoma japonica* is endemic in China and other countries of Asia, such as in Indonesia and the Philippines. Among 12 provinces of China (including municipalities and autonomous regions) that are endemic for *S*. *japonica*, Yunnan, Jiangsu, Hubei, Anhui, Jiangxi, and Hunan provinces achieved transmission control by the end of 2017. There are 450 endemic counties (including cities and districts) covering 259 million people in the country, specifically including 28,544 endemic villages with 70 million people at risk for contracting schistosomiasis [2].

The freshwater snail *Oncomelania hupensis* is the only intermediate host of *S*. *japonica*, the agent of the most virulent form of schistosomiasis [3–4]. The extermination of snails is an efficient method to control schistosomiasis, by eliminating snail transmission in the life cycle of schistosome parasites [5–6]. However, *O*. *hupensis*, is distributed throughout areas along the Yangtze (Yangzhe) River in China, including ditches, paddy fields, and bottomland of rivers and lakes. According to statistics in 2017, the area with a presence of *O*. *hupensis* snails in China was 172,501.56 hm^2^, of which 208.54 hm^2^ were areas with newly discovered populations of *O*. *hupensis*; snails have been found distributed over areas covering entire villages [2]. Such a wide and complex distribution of snails along the Yangtze River Basin is one of the important factors hampering the elimination of schistosomiasis in China and one of the main causes for a reemergent epidemic situation [7].

At present, niclosamide is the only chemical molluscicide recommended by the World Health Organization [8], which is widely used in China [9]. Despite its strong molluscicidal effect, the drug is expensive, insoluble in water, and prone to resistance after long-term use; therefore, the development of novel molluscicidal agents is critical [10]. Additionally, large-scale use of chemical molluscicides is liable to cause environmental pollution and endanger the health of aquatic organisms and people [4]. Plant-based molluscicides are considered simple, inexpensive, and environmentally safe alternatives to chemical molluscicides [11]. The use of plant-based molluscicides, however, is limited because of their low molluscicidal activity compared with chemical agents.

*Macleaya cordata* (Willd.) R.Br., a perennial deciduous plant in the family Papaveraceae, is widely distributed in China and is extensively used in traditional Chinese medicine for the treatment of wounds, arthritis, rheumatism arthralgia, and infection with *Trichomonas vaginalis* [4, 12]. Our initial study demonstrated that the alkaloid component AN2 extracted from *M*. *cordata* leaves had strong molluscicidal activity against *O*. *hupensis* [4]. However, the AN2 content in *M*. *cordata* leaves is low, thereby limiting its usefulness in practice. Reports have demonstrated that the alkaloid content is highest in the fruits of *M*. *cordata* than in all other plant organs [13]. *Macleaya cordata* in fruits contains a variety of alkaloids, among which quaternary benzo[c]phenanthridine alkaloids (QBAs) are the most abundant [14–15]. QBAs display a wide spectrum of non-specific biological activities, such as antimicrobial, antifungal, anti-inflammatory [16–17], and anticancer [18] effects. At present, QBAs have been explored as bioantibiotics for use in livestock [19–20]. In the present study, we investigated the molluscicidal effects of QBAs isolated from *M*. *cordata* fruits. The purpose of the experiments were: 1) to determine whether QBAs from *M*. *cordata* fruits have molluscicidal effects against *O*. *hupensis* using bioassay; 2) to analyze the physiological toxicity mechanism of QBAs from *M*. *cordata* fruits according to changes in the activity of several important detoxification enzymes: glutathione S-transferase (GST), carboxylesterase (CarE), acid phosphatase (ACP), and alkaline phosphatase (AKP).

## Materials and methods

All animals and plants for the experiments were collected from public lands.

### *Oncomelania hupensis* (*O*. *hupensis*)

Adult *O*. *hupensis* snails (9–11 mm in length) were collected from farm fields in the rural Taihu countryside of Jingzhou in Hubei Province of China. Among these, healthy snails were selected by examination for the presence of cercariae shedding, to eliminate schistosome-infected snails [21]. The selected snails were kept in a laboratory at 20°C for 1 week before the experiments.

### Ethics statement

The animal use protocol (#2017–1004) was approved by the Animal Care Use Committee of Life Science School of the Hubei University. All experiments were conducted in accordance with the guidelines of China national standard《Laboratory Animal—Requirements of Environment and Housing Facilities》(GB 14925–2010) and local guidelines of Hubei province standards《Regulations on the Administration of Laboratory Animals in Hubei Province》(Hubei government order NO.50 (2005)

### Plants, extraction of QBAs

The fruits of *M*. *cordata* plants were collected in September from Nanwudang Forest Park Nature Reserve in Yinshan County of Hubei Province.

The main alkaloids with higher content in the fruits of *M*. *cordata* are quaternary benzo[c]phenanthridine alkaloids (sanguinarine and chelerythrine) and protopine alkaloids (protopine and ɑ-allocryptopine). The four alkaloids can be extracted by acid method, and then QBAs can be isolated by alkali precipitation [15]. Therefore, QBAs were extracted from *M*. *cordata* fruits according to a modified method following Wang’s [15] and Fan’s [22] approaches of acid extraction and alkali precipitation.

**Extraction of the total alkaloids from *M*. *cordata* fruits**: Dry, pulverized *M*. *cordata* fruit powder was weighed, and 1000 g was added to a hydrochloric acid (HCl) solution (pH 1.5) at 1:10 (m:v); this mixture was immersed in a water bath at 85°C for 3 h. The leachate was filtered, then leached with HCl and filtered again; and impregnated repeatedly for 3 to 4 times until the solution was negative detected by bismuth potassium iodide. The filtrates from three rounds of extraction were combined. The filtrate was adjusted to pH 10 by adding a solution of sodium hydroxide and left standing overnight. The precipitate was then centrifuged and dried by a vacuum freeze-drying method, via which we obtained the total alkaloids (a brown powder).

**Separation and bisulfate preparation of QBAs from *M*. *cordata* fruits:** 20 g of total alkaloid was weighed and was added to 400 ml of 95% ethanol, dissolved, and filtered. Concentrated sulfuric acid was dripped into the filtrate until red precipitate no longer appeared; the filtrate was left to stabilize and then filtered to collect the precipitate. The precipitate was vacuum freeze-dried and a red powdery substance was obtained, which was the extracted QBAs (bisulfates).

### Determination of the main constituents and content of QBAs in *M*. *cordata* fruits

The main constituents and content of *M*. *cordata* QBAs were analyzed according to a modified method following Wang’s approach [15].

**Preparation of standard solution**: 40 mg sanguinarine (SA) and 20 mg chelerythrine (CHE) standards were weighed accurately (accurate to 0.04 mg) and (accurate to 0.02 mg), respectively, and placed in a 50 mL volumetric flask, respectively. A 50 mL solution of methanol and 1.0% HCl in 25:25 (v:v) ratio was added to the standards, ultrasonic dissolution and volume setting. 10 mL dissolution was accurately taken and placed in a 100 mL volumetric bottle, then the volume is fixed with a solution of methanol and 1.0% HCl (v:v = 50:50).

**Sample preparation and testing**: A total 20 mg of QBAs extract was weighed in a conical bottle and dissolved in a 100 mL solution of methanol and 1.0% HCl. Methanol and hydrochloric acid were added at 50:50 (v:v) and then weighed again. The QBAs solution was treated by ultrasound (power 250 W, 33 kHz). After 30 min, the solution was removed, cooled, and weighed once more. The lost mass was compensated by adding the abovementioned solvent mixture. The sample solution was shaken well, set aside for 30 minutes, and then filtered.

The sample filtrate and standards were respectively analyzed using high performance liquid chromatography–mass spectrometry (HPLC–MS/MS) (TSQ Quantum Access MAX, Thermo Fischer Scientific, USA) to obtain chromatographic and mass spectrometric data, respectively. The content of sanguinarine (SA) and chelerythrine bisulfate (CHE) in the samples was calculated by peak area according to an external standard method [15].

### Testing for molluscicidal activity

QBAs extracted as described above were dissolved in distilled water, and six treatments (0, 1 mg/L, 2.5 mg/L, 5 mg/L, 7.5 mg/L, and 10 mg/L) were administered to test molluscicidal activity of the QBAs. The selected snails were collected in a nylon mesh bag (20 snails per bag) and immersed in a 2-L red plastic bucket containing a 1-L solution with a known concentration of QBAs extract. Five bags of snails were exposed to each treatment for 24 h, 48 h, 72 h, 96 h, and 120 h, respectively, with exposure to dechlorinated tap water as the control treatment. To check snail mortality, no response to a needle probe under a dissecting microscope was considered evidence of death. The experiments were conducted in an incubator at 25°C, and each experiment was replicated three times. The lethal concentrations (LC_50_ and LC_90_) of QBAs against *O*. *hupensis* were calculated using IBM SPSS version 21 software (IBM Corp., Armonk, NY, USA).

### Assay of enzyme activity

According to the results of experiments to test molluscicidal activity described above, we used 72 h LC_50_ in an experiment to assess enzyme activity. Two hundred snails were randomly divided into four groups and exposed to four QBAs concentration levels (0, 1/4 LC_50_, 1/2 LC_50_, and LC_50_) for 24 h, 48 h, 72 h, 96 h, and 120 h, respectively, with dechlorinated water as the control. After treatment, one bag of snails was randomly selected from each group; the snails were washed with clean chlorine-free water, and the snail shell was lightly crushed. Liver tissue was subsequently excised, washed 2–3 times with chilled normal saline, and blotted dry on bibulous paper for biochemical analysis. Normal saline was added (3 mL/g of liver tissue) to the liver samples, which were then homogenized in an ice bath. The homogenates were then centrifuged at 8000 rpm for 10 min at 4°C. The supernatant was stored at 0–4°C for measurement of enzyme activity. The experiments were replicated three times under the same conditions.

Enzyme activity was measured according to the enzyme kinetic assay method of Guilbault et al. [23]. Enzyme activity was expressed as the amount of substrate hydrolyzed or production liberated in 1 mol/min/g protein in the supernatant. The total protein level of supernatants was estimated according to Bradford's method [24].

GST, CarE, AKP, and ACP kits were purchased from Shanghai AILEX Technology Co., Ltd. AKP activity was determined at 405 nm with 4-nitrophenyl phosphate as the substrate, and ACP activity was determined at 510 nm. The activities of GST and CarE were determined at 340 nm and 450 nm, respectively.

### Data analysis

The mortality rate in snails was expressed as the mean of three replicated experiments. The effect of QBAs against *O*. *hupensis* was expressed as LC_50_ and LC_90_ and their 95% confidence intervals. The results of enzyme activities were expressed as mean ± standard error (SE) of the three replicates. One-way analysis of variance (ANOVA) and simple sequence repeat (SSR, Duncan's repeat comparison) were used to detect significant differences (*P* < 0.05).

## Results

### Molluscicidal activity of QBAs

In the experiments, the number of deaths among *O*. *hupensis* snails treated with QBAs increased with increased treatment concentration and prolongation of the treatment time, revealing a concentration-dependent and time-dependent effect (Table 1). When treated with 2.5 mg/L QBAs for 48 h and 72 h, mortality rates in *O*. *hupensis* snails were over 60% and 90%, respectively; when treated with 10 mg/L QBAs for 24 h and 48 h, these rates were also over 60% and 90%, respectively. Compared with niclosamide at the same concentration (1 mg/L), we found that molluscicidal activity of QBAs against the snails was close to that of niclosamide (Table 1). These results showed that QBAs from *M*. *cordata* fruits had strong mollusicicidal activity.

**Table 1:** Molluscicidal activity of quaternary benzodiazepine alkaloids (QBAs) from *Macleaya cordata* fruits on *Oncomelania hupensis*.

Treatment	Concentration	Mortality (%)				
		24 h	48 h	72 h	96 h	120 h
Control		0.0 ± 0.00	1.7 ± 2.89	5.0 ± 0.00	6.7 ± 2.90	6.7 ± 2.90
QBAs	1 mg/L	8.3 ± 7.64	26.7 ± 2.89	31.7 ± 2.89	43.3 ± 2.90	48.3 ± 2.90
	2.5 mg/L	16.7 ± 2.89	61.7 ± 5.77	91.7 ± 2.89	98.3 ± 2.90	100.0 ± 0.00
	5 mg/L	33.3 ± 5.77	68.3 ± 7.64	95.0 ± 5.00	100.0 ± 0.00	100.0 ± 0.00
	7.5 mg/L	45.0 ± 8.66	91.7 ± 2.89	100.0 ± 0.00	100.0 ± 0.00	100.0 ± 0.00
	10 mg/L	63.3 ± 5.77	96.7 ± 2.89	100.0 ± 0.00	100.0 ± 0.00	100.0 ± 0.00
Niclosamide	1 mg/L	0.0 ± 0.00	30.0 ± 8.66	36.7 ± 7.64	58.3 ± 7.60	66.7 ± 7.60

Note: All data in the table are mean of three replicates ± SE.

### Enzyme activity

**Fig 1:**
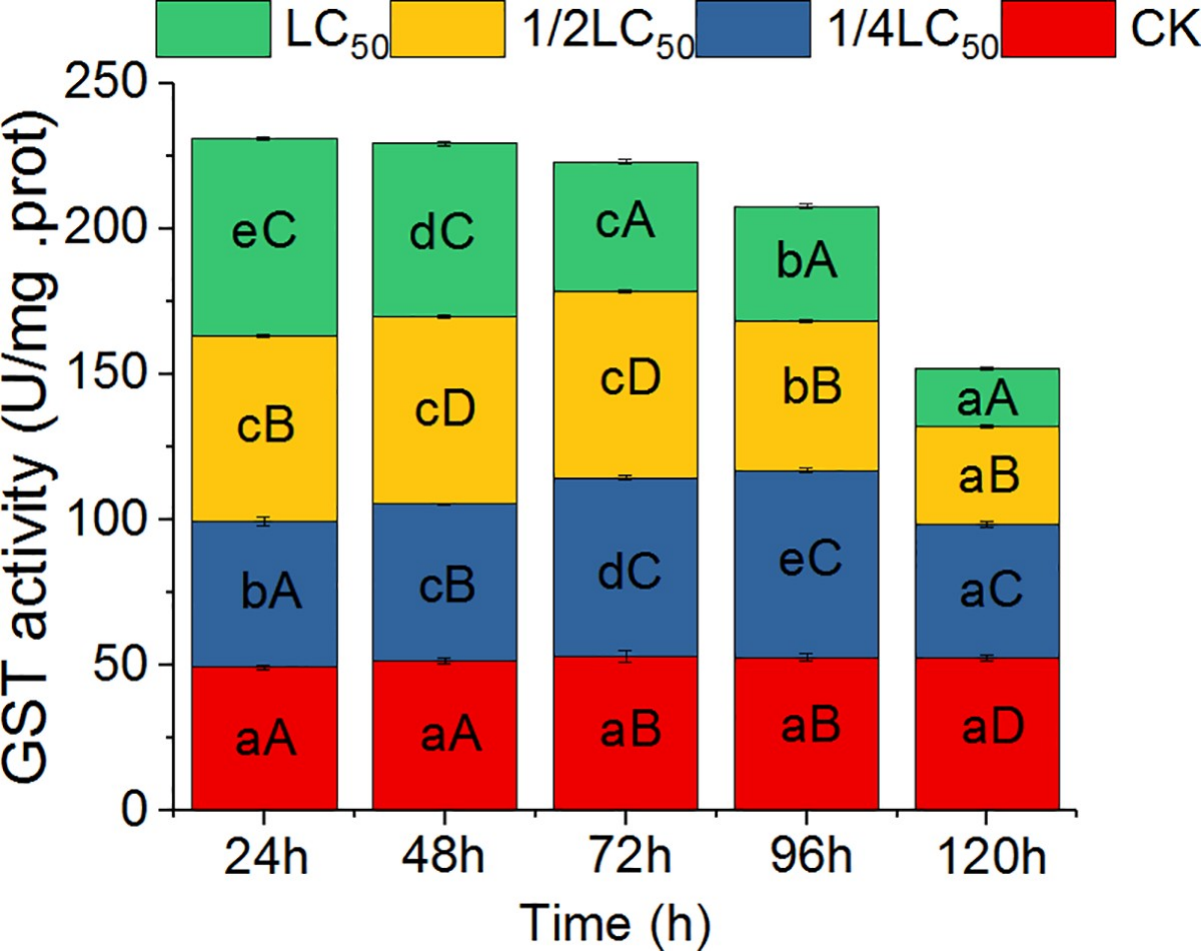
Effects of quaternary benzo[c]phenanthridine alkaloids from *Macleaya cordata* fruits on glutathione S-transferase (GST) activity in the liver of *Oncomelania hupensis* snails. Note: Different lowercase letters in the same color grid of different columns indicate significant differences at the same concentration with different treatment times; different uppercase letters in different color grids of the same column indicate significant differences at different concentrations with the same treatment times (*P* < 0.05). CK: Control.

In vivo, the activities of GST in the liver of snails treated with different concentrations of QBA increased significantly in the early stages of treatment (24 h), but decreased significantly in later treatment stages (Fig 1). For example, in treatment with higher concentrations of QBAs (1/2 LC_50_ and 1 LC_50_) for 24 h, the activities of GST increased sharply to 63.71 and 67.99 U/mg protein, respectively, which were significantly higher than that of the control (*P* < 0.05); however, there was no significant difference between treatment with 1/4 LC_50_ and the control. In treatment with 1/4 LC_50_, 1/2 LC_50_, and 1 LC_50_ for 120 h, GST activities were 45.63, 33.73 and 19.90 U/mg protein, which were significantly lower than that of the control (*P* < 0.05). Compared with the control, GST activity decreased by 13.5%, 36.1%, and 61.8%, respectively. With prolongation of treatment time, the enzyme activity responses differed, but all treatments showed the phenomenon of first being stimulated and then inhibited (Fig 1). For example, with low-concentration treatment (1/4 LC_50_), the peak of GST activity appeared at 96 h; with 1/2 LC_50_ and 1 LC_50_ treatments, the peaks appeared at 48 h and at 24 h, respectively.

**Fig 2:**
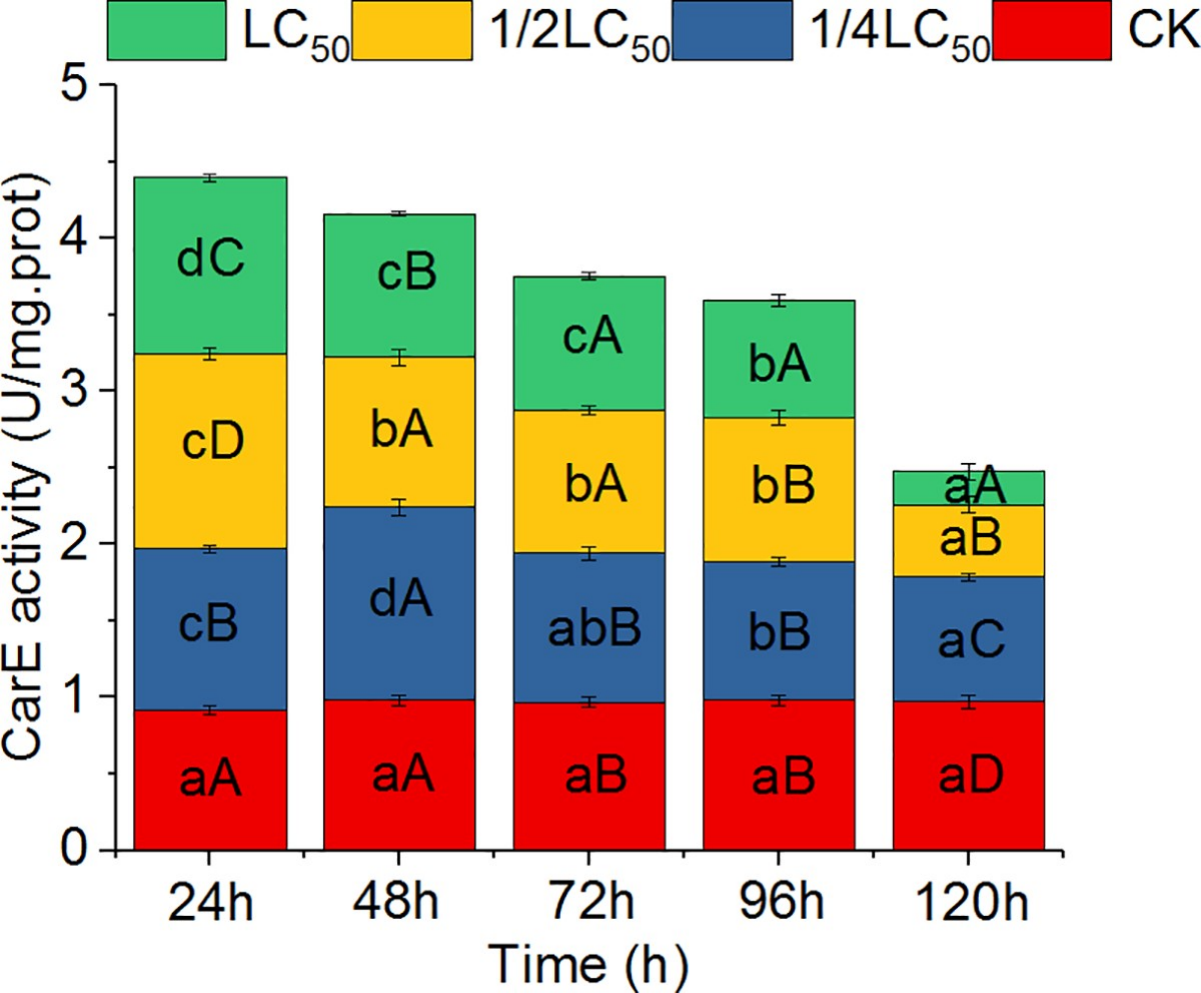
Effects of quaternary benzo[c]phenanthridine alkaloids from *Macleaya cordata* fruits on carboxylesterase (CarE) activity in the liver of *Oncomelania hupensis* snails. Note: Different lowercase letters in the same color grid of different columns indicate significant differences at the same concentration with different treatment times; different uppercase letters in different color grids of the same column indicate significant differences at different concentrations with the same treatment times (*P* < 0.05). CK: Control.

In this study, CarE activity in the liver of *O*. *hupensis* was significantly stimulated with all three concentrations of QBA in the early treatment stages (24 h) and was inhibited at later stages (120 h) (Fig 2). For example, CarE activities were 1.05, 1.27, and 1.15 U/mg protein after treatment at the three concentrations for 24 h, which were increased by 14.9%, 38.9%, and 25.5%, respectively, compared with the control. The CarE activities in the liver of snails treated for 120 h were 0.81, 0.47, and 0.21 U/mg protein, which had decreased to 83.7%, 48.6%, and 21.9% of the control level, respectively. With prolongation of treatment time, CarE activity decreased sharply; however, the enzyme activity with low-concentration treatment (1/4 LC_50_) continued to rise, with a peak at 48 h (Fig 2).

**Fig 3:**
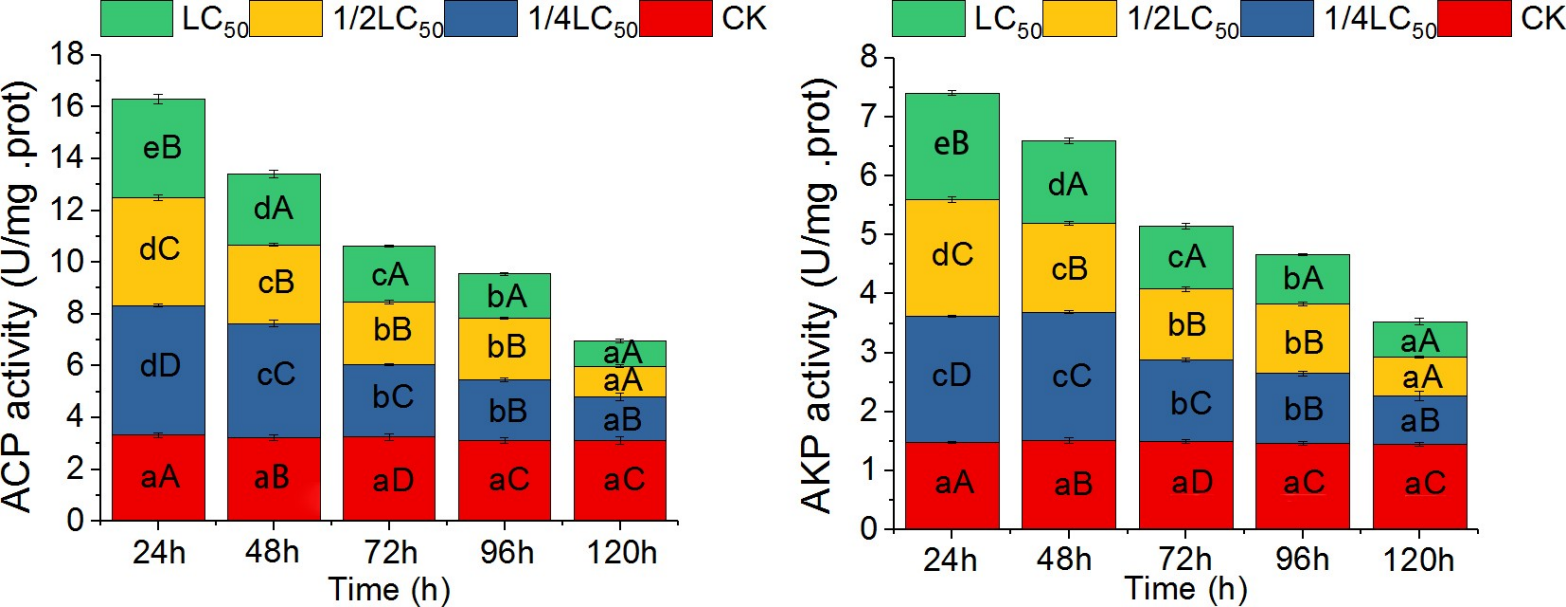
Effects of quaternary benzo[c]phenanthridine alkaloids from *Macleaya cordata* fruits on acid phosphatase (ACP; A) and alkaline phosphatase (AKP; B) activity in the liver of *Oncomelania hupensis* snails. Note: Different lowercase letters in the same color grid of different columns indicate significant differences at the same concentration with different treatment times; different uppercase letters in different color grids of the same column indicate significant differences at different concentrations with the same treatment times (*P* < 0.05). CK: Control.

Compared with the control, we found that the activities of AKP and ACP in the liver of *O*. *hupensis* increased significantly in treatment with the three concentrations of QBAs for 24 h (Fig 3A and 3B). However, the extent of increase declined gradually with increased concentration, which showed an opposite concentration-dependent effect to that of GST (Fig 1). With the prolongation of treatment time, AKP and ACP activities decreased sharply; however, ACP activity with low-concentration treatment (1/4 LC_50_) continued to rise, with a peak at 48 h (Fig 3B), similar to that of CarE. The degree of decline grew larger with increased treatment concentration. From 72 h, the enzyme activity was significantly lower than that of the control.

## Discussion

At present, a variety of alkaloids have been found in the whole plant of *M*. *cordata* [16, 25–26]. The main alkaloids with the highest content in *M*. *cordata* are QBAs and protopine-type alkaloids, which are important groups in the isoquinoline alkaloid family [17–18]; The highest concentration of QBAs can be found in the fruits of *M*. *cordata* [13]. It has been demonstrated that QBAs have anti-bacterial, anti-inflammatory, anti-tumor, analgesic, diuretic, and other effects [15–20, 26]. However, there are few reports on molluscicidal activities in the snail *O*. *hupensis* [4, 27]. The results in our study showed that QBAs extracted from *M*. *cordata* fruits had strong molluscicidal activity against these snails. The LC_50_ and LC_90_ values were 1.29 mg/L (95% confidence internal: 0.507–2.005 mg/L) and 2.92 mg/L (95% confidence internal: 1.821–3.990 mg/L) at 72 h, respectively, which were higher than those of cardiac glycosides extracted from *Nerium indicum* (LC_50_ 4.05 mg/L and LC_90_ 22.25 mg/L) [28] and the alkaloid AN2 in *M*. *cardata*, which was previously found in our laboratory [4]. The molluscicidal activity of QBAs is also close to that of the chemical molluscicide niclosamide (Table 1). Although the molluscicidal activity of QBAs from the *M*. *cordata* plant is lower than that of the standard chemical molluscicide niclosamide, QBA fractions from *M*. *cordata* are often used in toothpastes and mouthwashes as antiplaque agents, are applied as antifungal and anti-inflammatory preparations in Russia [17], and are even used as alternative antibiotic feed additives in other countries [20, 29]. Therefore, QBAs as plant molluscicides are substantially safer for human beings and the environment [4].

**Fig 4:**
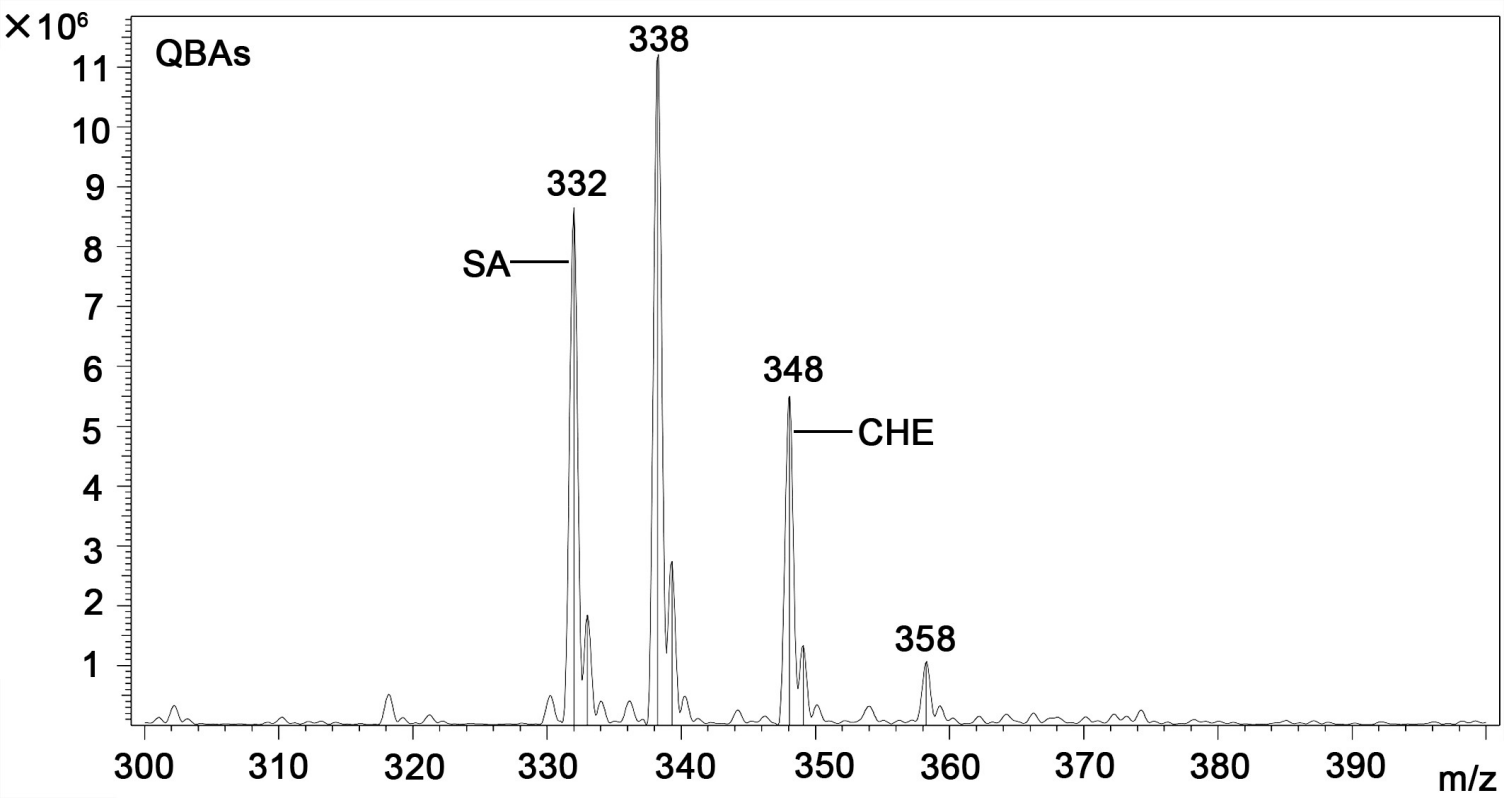
Chromatogram of quaternary benzo[c]phenanthridine alkaloids in *Macleaya cordata* fruits determined by HPLC-MS. Note: The peak at 338 was an impurity peak in the HPLC-MS spectrometer.

**Fig 5:**
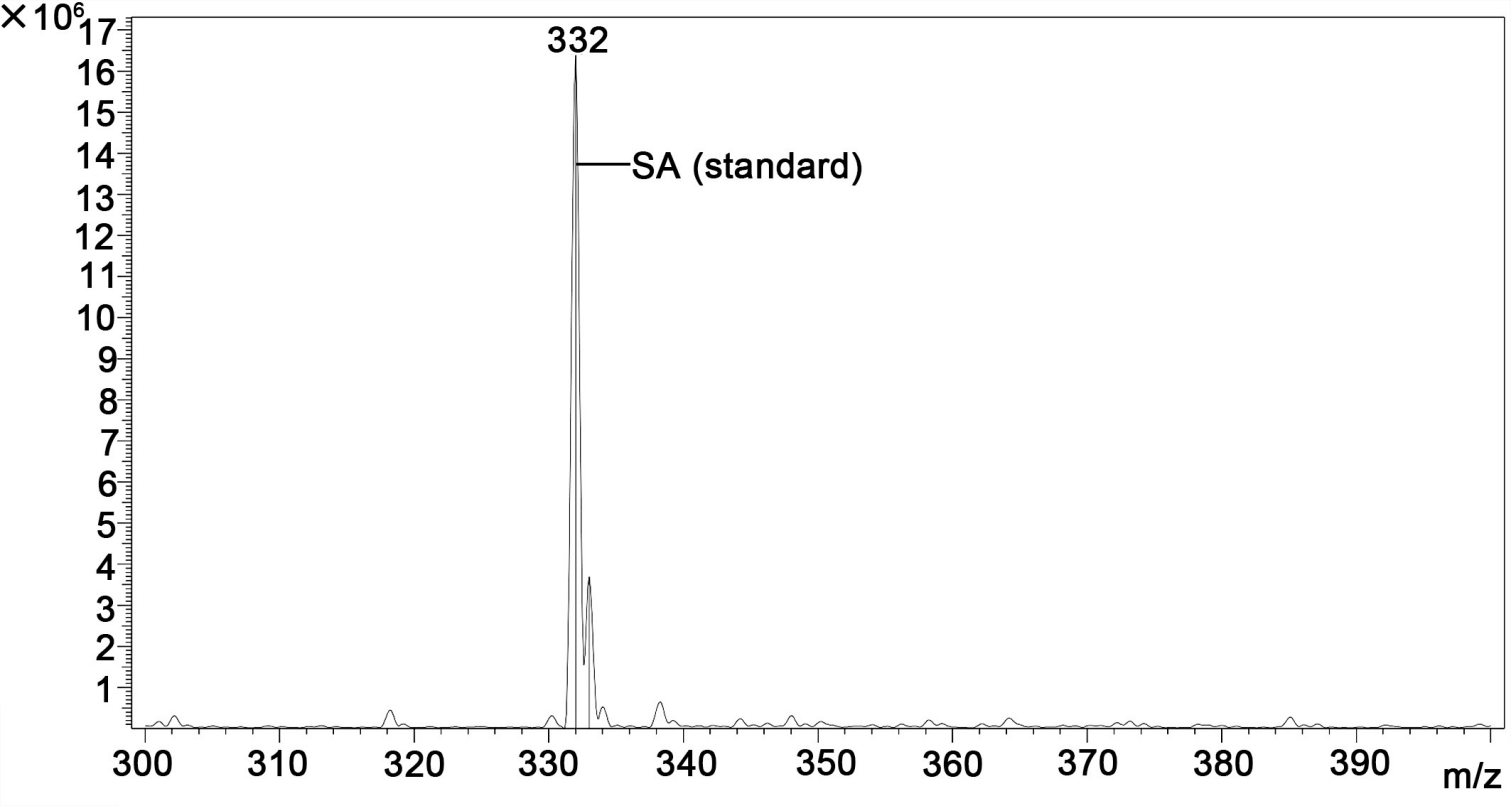
Chromatogram of sanguinarine (SA) standard sample determined by HPLC-MS.

**Fig 6:**
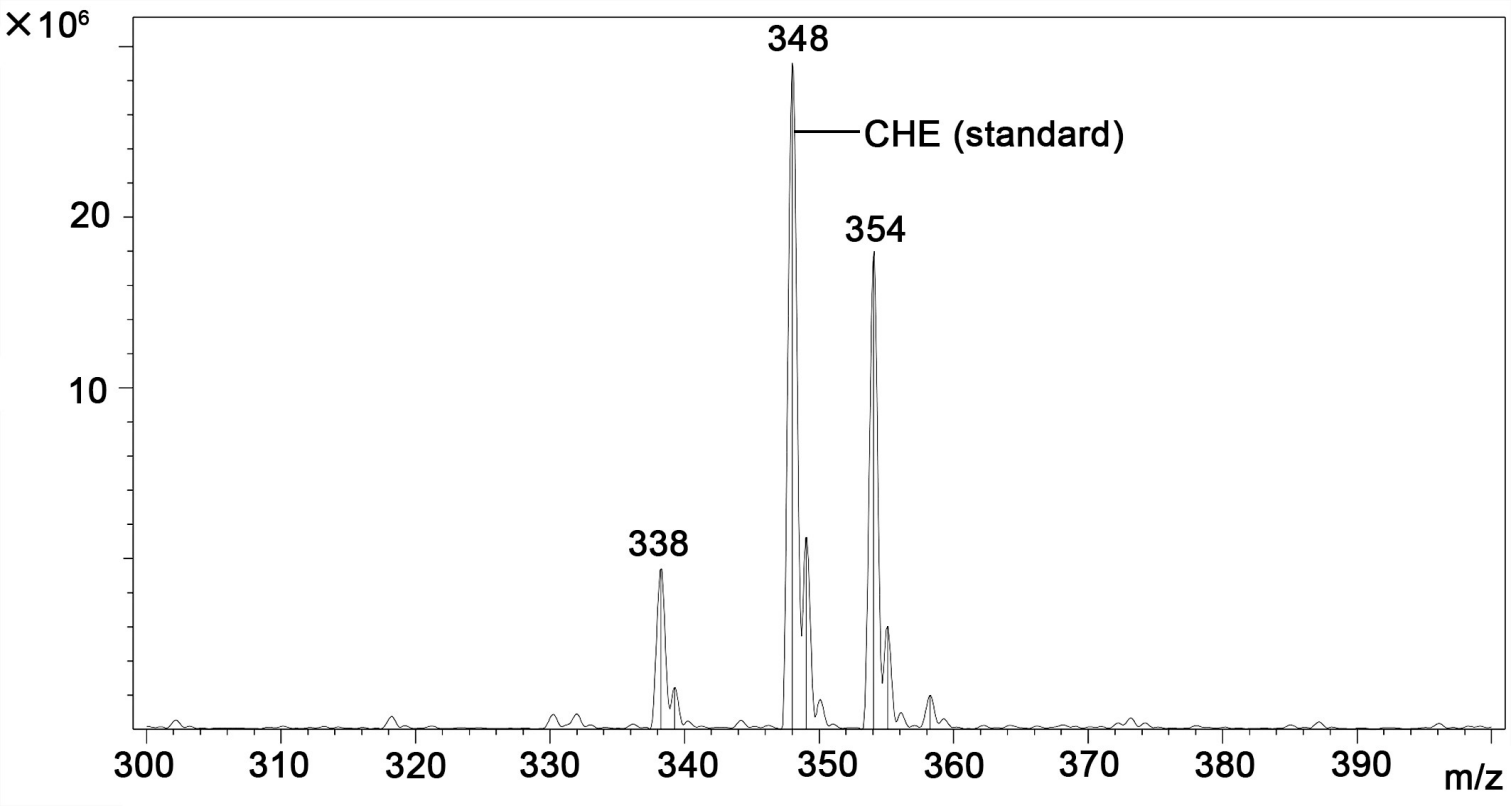
Chromatogram of chelerythrine (CHE) standard sample determined by HPLC-MS.

**Fig 7:**
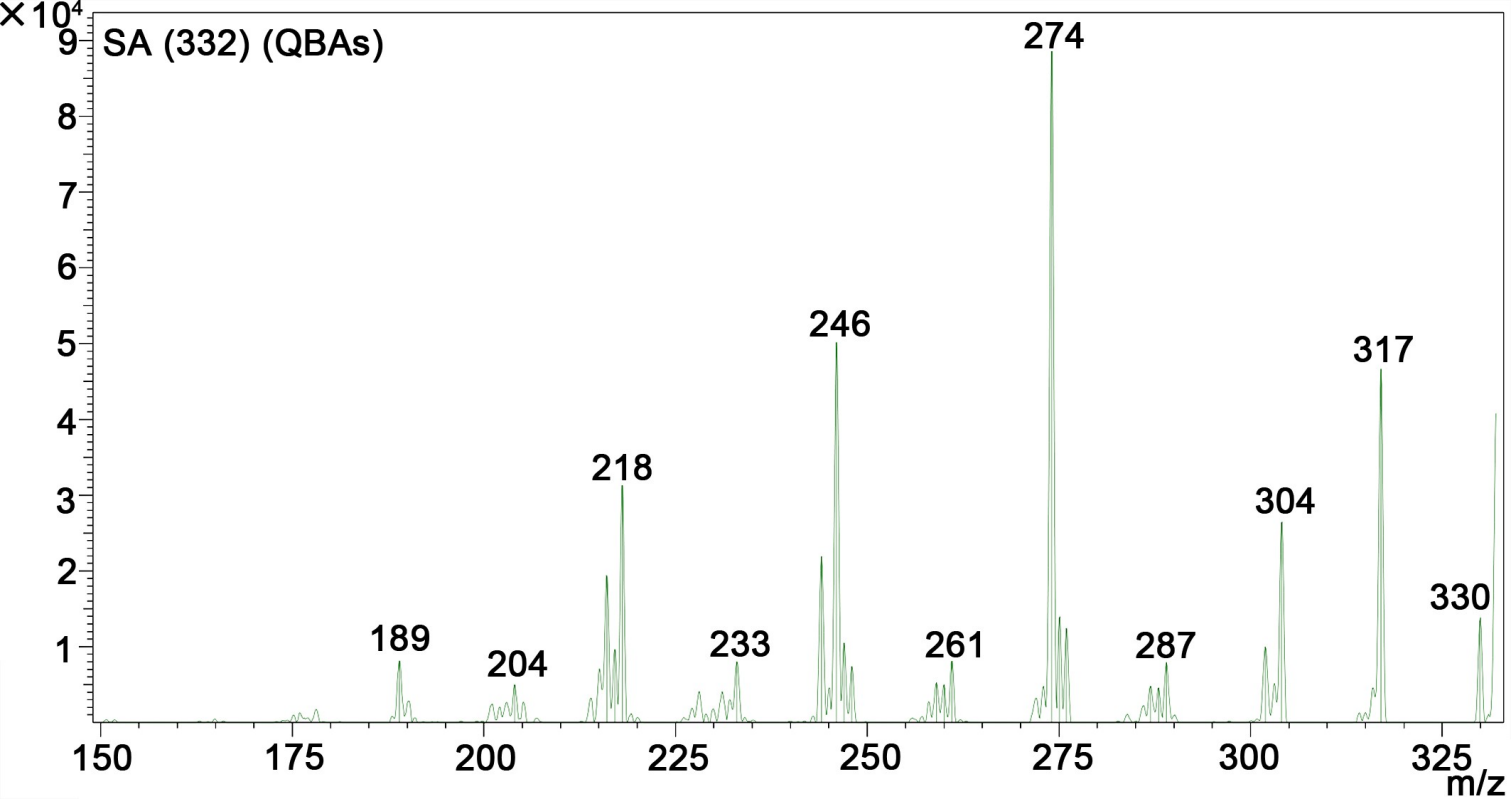
Mass spectrogram of quaternary benzo[c]phenanthridine alkaloids (BasePeak:332).

**Fig 8:**
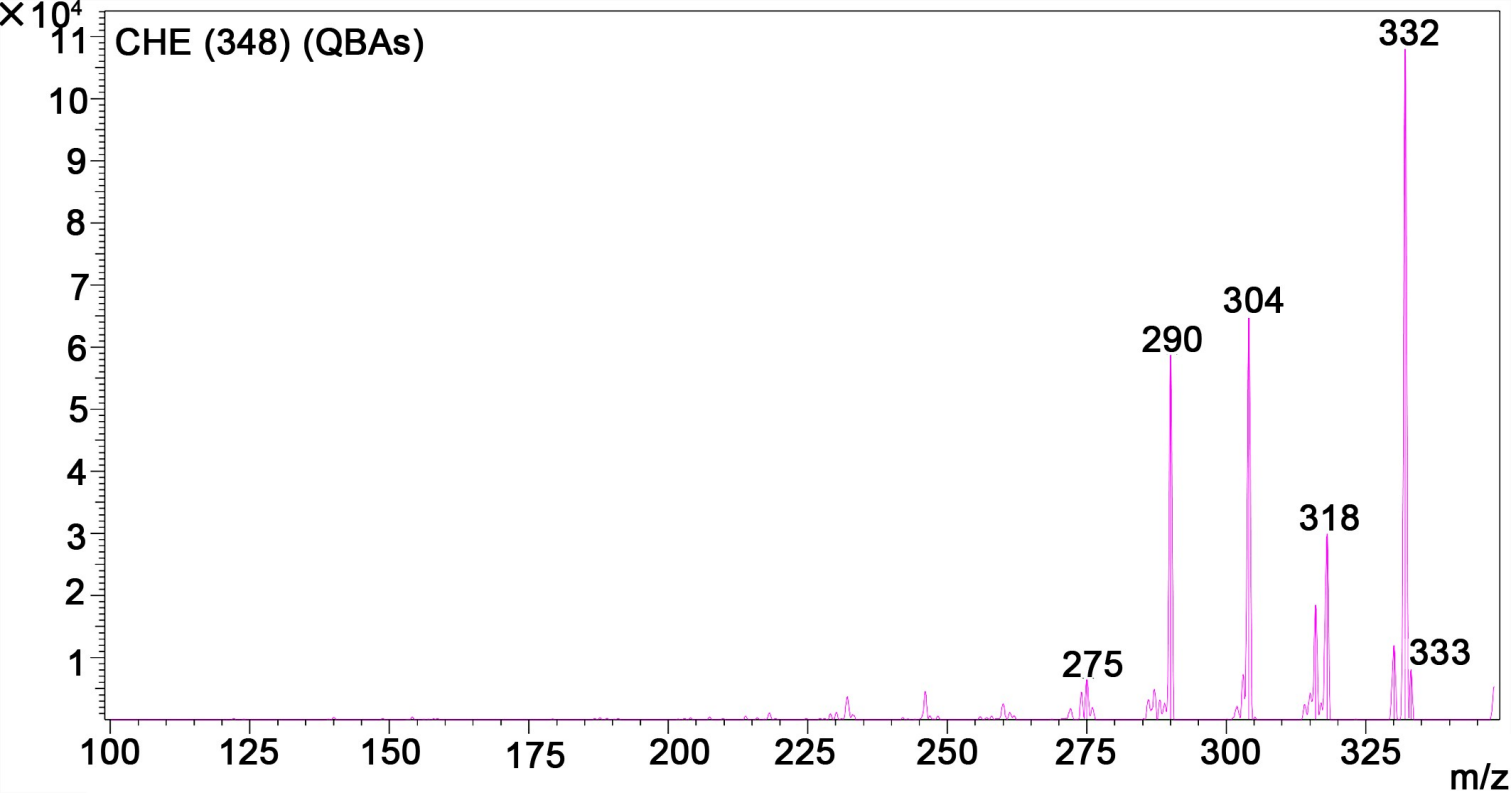
Mass spectrogram of quaternary benzo[c]phenanthridine alkaloids (BasePeak:348).

**Fig 9:**
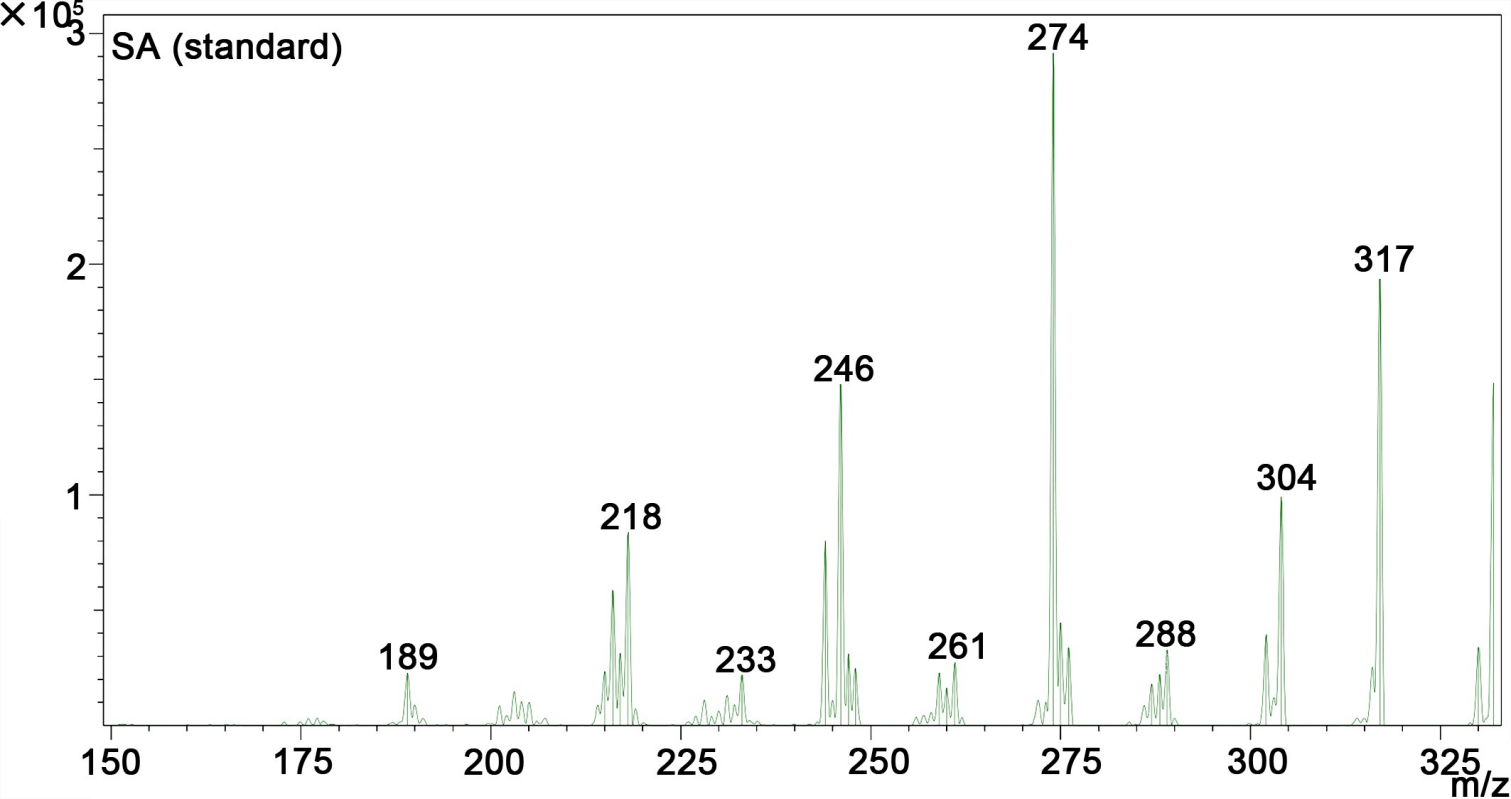
Mass spectrogram of sanguinarine (SA) standard sample.

**Fig 10:**
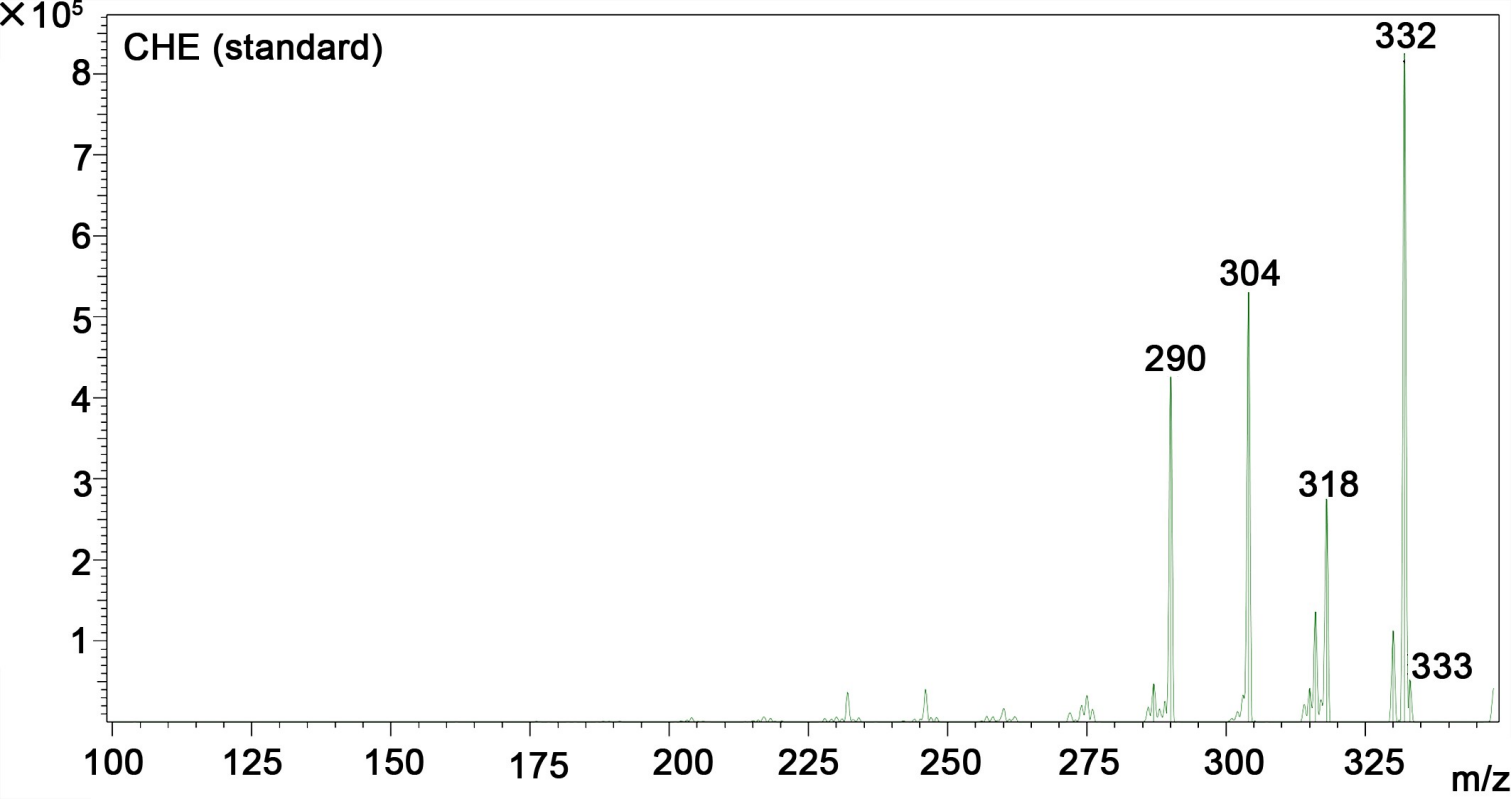
Mass spectrogram of chelerythrine (CHE) standard sample.

A quantitative study including HPLC-MS analysis showed that QBAs from the fruits of *M*. *cordata* contained two main basic peaks 332 and 348 (Fig 4). Compared with standard samples of sanguinarine (SA) and chelerythrine (CHE) (Figs 5 and 6), the results revealed that the QBAs extracted from *M*. *cordata* fruits mainly contained SA and CHE, accounting for 43.4% and 19.05% of total QBAs, respectively. Analyzed by mass spectrometry, the molecular ion peaks of these two basic peaks were MS (m/z): 274 [M^+^] and 332 [M^+^] (Figs 7 and 8), respectively, which were consistent with the molecular ion peaks of SA and CHE standards (Figs 9 and 10). The results were further revealed that the two main components of QBAs were SA and CHE. Some reports have also showed that SA and CHE are the two main QBAs found in the capsule of *M*. *cordata* [14, 16]. In practical application in the field, it has been found that niclosamide is inconvenient to use because it is insoluble in water, thus limiting its molluscicidal effect. QBAs (disulfate) in our study were extracted in the form of a reddish brown powder that is soluble in water, which is far more convenient in practical application.

Some compounds might affect vital enzyme activities and lead to death in snails [30]. In the present experiments, the GST activity in the liver of snails treated with the extracted QBAs increased significantly with increased QBA concentration in early treatment phases (e.g., 24 h; *P* < 0.05); however, GST activity was significantly decreased by the end of treatment (120 h) at all concentrations and was obviously lower than that of the control (Fig 1; *P* < 0.05). This phenomenon of intensive increase followed by intensive decline has also been observed in insects and other animals treated with active plant ingredients. For example, GST activity in *Agasicles hygrophila* and *Helicoverpa armigera* could be stimulated by quercetin, tannic acid, orange tertiary alcohol, and oleanolic acid with shorter treatment times at low doses, but this activity was inhibited with longer times at high doses [31–32]. GSTs belong to a multigene family of dimeric, multifunctional proteins that have a central role in detoxification of xenobiotic compounds including drugs, herbicides, and insecticides [33–34]. These enzymes catalyze nucleophilic attack by reduced glutathione (GSH) on nonpolar compounds. In our study, during early phases of QBA treatment, the increase in GST activity was correlated with emergency responses in the snail. At the end of treatment, however, the dramatic decrease in GST activity implies decreased detoxification function in the liver of the snail, which could result in death among *Oncomelania*.

CarE is mainly located in the liver and other tissues. As one of the most important metabolic detoxification enzymes, CarE has been well studied in insects [35–36] and mollusks [37–39]. In our experiments, the CarE activity in the liver tissue of *Oncomelania* increased significantly in early QBA treatment (Fig 2; 24 h). This might be owing to an emergency response induced by the QBAs, which implies that CarE in the liver of the snails was detoxifying the QBAs. This induction has been reported in many studies on the effects of pesticides on insect detoxification enzymes [40–42]. With prolonged treatment time and increased concentration, however, CarE activity decreased sharply and was significantly lower than that of the control at later treatment stages, such as at 120 h (Fig 2). This significant inhibition of CarE indicated that the ability of detoxification and metabolism in the liver of *Oncomelania* snails decreased sharply, leading to accumulation of toxicants in vivo and death owing to poisoning. Moreover, this type of inhibition may be noncompetitive inhibition [43].

AKP and ACP are ubiquitous in animals, plants, and microorganisms. These enzymes are directly involved in the process of phosphate group transfer and metabolism in organisms and therefore have an important role in the metabolism of substances [44]. Additionally, as an important component of lysosomal enzymes in mollusks, these enzymes play an important part in the immune response [45–47]. In our experiments, we found that QBAs stimulated a significant increase in AKP and ACP activity in the liver of *O*. *hupensis* snails at all three concentrations in early stages of treatment. But the activities of the two enzymes decreased with increased QBA concentration. With prolongation of treatment time, the activity of AKP had decreased sharply by the end of QBA treatment (120 h) and was significantly lower than that of the control (Fig 3). In mollusks, lysosomal enzymes generally have the dual functions of defense and digestion. Hydrolysis of lysosomal enzymes has been identified as one of the main mechanisms for the organism to attack foreign bodies [47]. AKP and ACP are two important components of lysosomal enzymes in mollusks; in particular, ACP is a marker of lysosomal enzymes. The significant increases of AKP and ACP in the early stages of treatment in our experiments indicate that the metabolic activity of hepatic lysosomes in *Oncomelania* and the attack digestion of foreign QBAs were critically strengthened. However, with increased QBA concentration and the accumulation of hepatocytes, AKP and ACP in lysosomes were inhibited, their activities decreased sharply, and the digestive and metabolic functions with respect to exogenous QBAs were reduced, thus reducing the detoxification functions in the snail liver. At the same time, lysosomes have a role in the immune response [44–47]; therefore, QBAs may also destroy hemocytes and lymphocytes in *O*. *hupensis*, thus affecting the snail’s immune function.

### Conclusion

The plant *M*. *cordata* is widely distributed in China and is often used in traditional Chinese medicine. Our research results showed that QBAs extracted from *M*. *cordata* fruits had strong molluscicidal activity against *O*. *hupensis*. Therefore, QBAs have potential development value as a biological molluscicide, which could provide an environmentally friendly alternative to help solve the problem of environmental contamination owing to chemical molluscicides. The main active ingredients of QBAs are SA and CHE, the ratio of which is about 2:1. QBAs (48 h LC_50_ value was 2.89 mg/L) comprising a natural combination of SA and CHE had stronger molluscicidal activity than SA alone (48 h LC_50_ value was 6.35 mg/L). This natural composition of QBAs could provide a scientific basis for the development of biomolluscicides using these active ingredients and proportions in the future.

In this study, changes in the activities of the metabolic detoxification enzymes GST, CarE, AKP, and ACP in the liver of *O*. *hupensis* showed that the mechanism of death in these snails caused by QBAs might be related to the effect of sharply reducing some metabolic detoxification functions, immune functions, and other metabolic functions such as energy metabolism.

## Supporting information

S1 Table Molluscicidal activity of quaternary benzodiazepine alkaloids (QBAs) from *Macleaya cordata* fruits on *Oncomelania hupensis*. (DOC)

S1 Fig Effects of quaternary benzo[c]phenanthridine alkaloids from *Macleaya cordata* fruits on glutathione S-transferase (GST) activity in the liver of *Oncomelania hupensis* snails. One-way analysis of variance (ANOVA) and simple sequence repeat (SSR, Duncan's repeat comparison of **glutathione S-transferase (GST) data.**
(DOC)

S2 Fig Effects of quaternary benzo[c]phenanthridine alkaloids from Macleaya cordata fruits on carboxylesterase (CarE) activity in the liver of Oncomelania hupensis snails. One-way analysis of variance (ANOVA) and simple sequence repeat (SSR, Duncan's repeat comparison of **carboxylesterase (CarE) data.**
(DOC)

S3 Fig Effects of quaternary benzo[c]phenanthridine alkaloids from *Macleaya cordata* fruits on acid phosphatase (ACP; A) activity in the liver of *Oncomelania hupensis* snails. One-way analysis of variance (ANOVA) and simple sequence repeat (SSR, Duncan's repeat comparison of acid phosphatase (ACP) data (Fig 3A). Effects of quaternary benzo[c]phenanthridine alkaloids from *Macleaya cordata* fruits on alkaline phosphatase (**AKP; B**) activity in the liver of *Oncomelania hupensis* snails. One-way analysis of variance (ANOVA) and simple sequence repeat (SSR, Duncan's repeat comparison of **alkaline phosphatase (AKP) data(Fig 3B).**
(DOC)

S4 Fig Chromatogram of quaternary benzo[c]phenanthridine alkaloids in *Macleaya cordata* fruits determined by HPLC-MS (Fig 4) and two standard samples (sanguinarine (SA) and chelerythrine (CHE) (Fig 5 and Fig 6). (DOC)

S5 Fig Mass spectrogram of quaternary benzo[c]phenanthridine alkaloids (Fig 7 and Fig 8) two standard mass spectrum (Fig 9 and Fig 10). (DOC)
