# Supplementary material for: Molluscicidal activity and physiological toxicity of quaternary benzo[c]phenanthridine alkaloids (QBAs) from Macleaya cordata fruits on Oncomelania hupensis
Source: PLoS Negl Trop Dis. 2019 Oct 11;13(10):e0007740. doi: 10.1371/journal.pntd.0007740 (PMC6808491; doi:10.1371/journal.pntd.0007740)
Supplement: S1 Table — (DOC) [file pntd.0007740.s001.doc]

**Table 1 Molluscicidal activity of quaternary benzodiazepine alkaloids (QBAs) from *Macleaya cordata* fruits On *Oncomelania hupensis***

| Treatment | Concentration | Mortality (%) | | | | |
| --- | --- | --- | --- | --- | --- | --- |
|  |  | 24 h | 48 h | 72 h | 96 h | 120 h |
| Control |  | 0.0 ± 0.00 | 1.7 ± 2.89 | 5.0 ± 0.00 | 6.7 ± 2.90 | 6.7 ± 2.90 |
| QBAs | 1 mg/L | 8.3 ± 7.64 | 26.7 ± 2.89 | 31.7 ± 2.89 | 43.3 ± 2.90 | 48.3 ± 2.90 |
|  | 2.5 mg/L | 16.7 ± 2.89 | 61.7 ± 5.77 | 91.7 ± 2.89 | 98.3 ± 2.90 | 100.0 ± 0.00 |
|  | 5 mg/L | 33.3 ± 5.77 | 68.3 ± 7.64 | 95.0 ± 5.00 | 100.0 ± 0.00 | 100.0 ± 0.00 |
|  | 7.5 mg/L | 45.0 ± 8.66 | 91.7 ± 2.89 | 100.0 ± 0.00 | 100.0 ± 0.00 | 100.0 ± 0.00 |
|  | 10 mg/L | 63.3 ± 5.77 | 96.7 ± 2.89 | 100.0 ± 0.00 | 100.0 ± 0.00 | 100.0 ± 0.00 |
| Niclosamide | 1 mg/L | 0.0 ± 0.00 | 30.0 ± 8.66 | 36.7 ± 7.64 | 58.3 ± 7.60 | 66.7 ± 7.60 |

Note: All data in the table are mean of three replicates ± SE.
