# Supplementary material for: Molluscicidal activity and physiological toxicity of quaternary benzo[c]phenanthridine alkaloids (QBAs) from Macleaya cordata fruits on Oncomelania hupensis
Source: PLoS Negl Trop Dis. 2019 Oct 11;13(10):e0007740. doi: 10.1371/journal.pntd.0007740 (PMC6808491; doi:10.1371/journal.pntd.0007740)
Supplement: S1 Fig — One-way analysis of variance (ANOVA) and simple sequence repeat (SSR, Duncan's repeat comparison of glutathione S-transferase (GST) data. (DOC) [file pntd.0007740.s002.doc]

**S2 fig. 1**

One-way analysis of variance (ANOVA) and simple sequence repeat (SSR, Duncan's repeat comparison of **glutathione S-transferase (GST) data**

**1.ONEWAY h24 h48 h72 h96 h120 BY C /STATISTICS DESCRIPTIVES /MISSING ANALYSIS /POSTHOC=DUNCAN ALPHA(0.05).**

Numbers of “1.00, 2.00, 3.00, 4.00” in concentration column of the tables mean the concentration of “Control, 1/4LC_50_, 1/2LC_50_ and LC_50_”, respectively.

| STATISTICS DESCRIPTIVES | | | | | | | | |  |
| --- | --- | --- | --- | --- | --- | --- | --- | --- | --- |
| Time(h)/Concentrations | | N | Mean Value | Standard deviation | Standard Error | 95% confidence interval for the mean | | Minimum | Maximum |
|  |  |  |  |  |  | Lower limit | Upper limit |  |  |
| h24 | 1.00 | 3 | 49.5233 | .80934 | .46727 | 47.5128 | 51.5338 | 48.67 | 50.28 |
|  | 2.00 | 3 | 49.9533 | 1.71419 | .98969 | 45.6951 | 54.2116 | 48.03 | 51.32 |
|  | 3.00 | 3 | 63.7233 | .57709 | .33318 | 62.2898 | 65.1569 | 63.12 | 64.27 |
|  | 4.00 | 3 | 67.7700 | .58026 | .33501 | 66.3286 | 69.2114 | 67.34 | 68.43 |
|  | Total | 12 | 57.7425 | 8.53957 | 2.46516 | 52.3167 | 63.1683 | 48.03 | 68.43 |
| h48 | 1.00 | 3 | 51.8233 | 1.05434 | .60872 | 49.2042 | 54.4425 | 51.08 | 53.03 |
|  | 2.00 | 3 | 53.7367 | .29738 | .17169 | 52.9979 | 54.4754 | 53.42 | 54.01 |
|  | 3.00 | 3 | 64.3000 | .44911 | .25929 | 63.1843 | 65.4157 | 63.89 | 64.78 |
|  | 4.00 | 3 | 59.4200 | .69505 | .40129 | 57.6934 | 61.1466 | 58.73 | 60.12 |
|  | Total | 12 | 57.3200 | 5.15507 | 1.48814 | 54.0446 | 60.5954 | 51.08 | 64.78 |
| h72 | 1.00 | 3 | 53.1400 | 1.97881 | 1.14247 | 48.2244 | 58.0556 | 51.87 | 55.42 |
|  | 2.00 | 3 | 61.3267 | .82403 | .47576 | 59.2797 | 63.3737 | 60.42 | 62.03 |
|  | 3.00 | 3 | 64.1033 | .61712 | .35629 | 62.5703 | 65.6363 | 63.42 | 64.62 |
|  | 4.00 | 3 | 44.4300 | .81615 | .47120 | 42.4026 | 46.4574 | 43.64 | 45.27 |
|  | Total | 12 | 55.7500 | 8.08354 | 2.33352 | 50.6140 | 60.8860 | 43.64 | 64.62 |
| h96 | 1.00 | 3 | 52.9100 | 1.38286 | .79839 | 49.4748 | 56.3452 | 51.62 | 54.37 |
|  | 2.00 | 3 | 64.1867 | .71710 | .41402 | 62.4053 | 65.9680 | 63.44 | 64.87 |
|  | 3.00 | 3 | 51.1300 | .45574 | .26312 | 49.9979 | 52.2621 | 50.61 | 51.46 |
|  | 4.00 | 3 | 39.4867 | .83524 | .48223 | 37.4118 | 41.5615 | 38.64 | 40.31 |
|  | Total | 12 | 51.9283 | 9.17830 | 2.64955 | 46.0967 | 57.7599 | 38.64 | 64.87 |
| h120 | 1.00 | 3 | 52.7667 | 1.06265 | .61352 | 50.1269 | 55.4064 | 51.75 | 53.87 |
|  | 2.00 | 3 | 45.6267 | .99420 | .57400 | 43.1569 | 48.0964 | 44.86 | 46.75 |
|  | 3.00 | 3 | 33.7333 | .74312 | .42904 | 31.8873 | 35.5794 | 33.15 | 34.57 |
|  | 4.00 | 3 | 19.8967 | .48014 | .27721 | 18.7039 | 21.0894 | 19.37 | 20.31 |
|  | Total | 12 | 38.0058 | 13.0463 | 3.76614 | 29.7166 | 46.2951 | 19.37 | 53.87 |

| **One-way ANOVA** | | | | | | |
| --- | --- | --- | --- | --- | --- | --- |
|  | | Quadratic sum | df | Mean square | F | Significance |
| h24 | Intergroup | 793.641 | 3 | 264.547 | 248.214 | .000 |
|  | Intragroup | 8.526 | 8 | 1.066 |  |  |
|  | Total | 802.167 | 11 |  |  |  |
| h48 | Intergroup | 288.552 | 3 | 96.184 | 204.118 | .000 |
|  | Intragroup | 3.770 | 8 | .471 |  |  |
|  | Total | 292.322 | 11 |  |  |  |
| h72 | Intergroup | 707.496 | 3 | 235.832 | 167.207 | .000 |
|  | Intragroup | 11.283 | 8 | 1.410 |  |  |
|  | Total | 718.779 | 11 |  |  |  |
| h96 | Intergroup | 919.988 | 3 | 306.663 | 368.157 | .000 |
|  | Intragroup | 6.664 | 8 | .833 |  |  |
|  | Total | 926.652 | 11 |  |  |  |
| h120 | Intergroup | 1866.466 | 3 | 622.155 | 858.017 | .000 |
|  | Intragroup | 5.801 | 8 | .725 |  |  |
|  | Total | 1872.267 | 11 |  |  |  |

**Similar subset after Duncan's repeat comparison**

| **h24** | | | | |
| --- | --- | --- | --- | --- |
| Duncan^a^ | | | | |
| C | N | A subset of alpha = 0.05 | | |
|  |  | 1 | 2 | 3 |
| 1.00 | 3 | 49.5233 |  |  |
| 2.00 | 3 | 49.9533 |  |  |
| 3.00 | 3 |  | 63.7233 |  |
| 4.00 | 3 |  |  | 67.7700 |
| Significance |  | .624 | 1.000 | 1.000 |

| Display the group mean in the same subset of the table | | | | | |  |
| --- | --- | --- | --- | --- | --- | --- |
| a. Harmonic mean sample size is used = 3.000 | | | | | |  |
| **h48** | | | | | | |
| Duncan^a^ | | | | | | |
| C | N | A subset of alpha = 0.05 | | | | |
|  |  | 1 | 2 | 3 | 4 | |
| 1.00 | 3 | 51.8233 |  |  |  | |
| 2.00 | 3 |  | 53.7367 |  |  | |
| 4.00 | 3 |  |  | 59.4200 |  | |
| 3.00 | 3 |  |  |  | 64.3000 | |
| Significance |  | 1.000 | 1.000 | 1.000 | 1.000 | |
| **h72** | | | | | | |
| Duncan^a^ | | | | | | |
| C | N | A subset of alpha = 0.05 | | | | |
|  |  | 1 | 2 | 3 | 4 | |
| 4.00 | 3 | 44.4300 |  |  |  | |
| 1.00 | 3 |  | 53.1400 |  |  | |
| 2.00 | 3 |  |  | 61.3267 |  | |
| 3.00 | 3 |  |  |  | 64.1033 | |
| Significance |  | 1.000 | 1.000 | 1.000 | 1.000 | |

| **h96** | | | | | |
| --- | --- | --- | --- | --- | --- |
| Duncan^a^ | | | | | |
| C | N | A subset of alpha = 0.05 | | | |
|  |  | 1 | 2 | 3 | 4 |
| 4.00 | 3 | 39.4867 |  |  |  |
| 3.00 | 3 |  | 51.1300 |  |  |
| 1.00 | 3 |  |  | 52.9100 |  |
| 2.00 | 3 |  |  |  | 64.1867 |
| Significance |  | 1.000 | 1.000 | 1.000 | 1.000 |
| **h120** | | | | | |
| Duncan^a^ | | | | | |
| C | N | A subset of alpha = 0.05 | | | |
|  |  | 1 | 2 | 3 | 4 |
| 4.00 | 3 | 19.8967 |  |  |  |
| 3.00 | 3 |  | 33.7333 |  |  |
| 2.00 | 3 |  |  | 45.6267 |  |
| 1.00 | 3 |  |  |  | 52.7667 |
| Significance |  | 1.000 | 1.000 | 1.000 | 1.000 |

| Display the group mean in the same subset of the table |
| --- |
| a. Harmonic mean sample size is used = 3.000 |

1. **ONEWAY CK C1 C2 C3 BY Time /STATISTICS DESCRIPTIVES /MISSING ANALYSIS /POSTHOC=DUNCAN ALPHA(0.05).**

|  | | | | | | | | |  |  |
| --- | --- | --- | --- | --- | --- | --- | --- | --- | --- | --- |
| Concentrations/ Time(h) | | N | Mean Value | Standard deviation | Standard Error | 95% confidence interval for the mean | | Minimum | Maximum |  |
|  |  |  |  |  |  | Lower limit | Upper limit |  |  |  |
| CK | 24.00 | 3 | 49.5233 | .80934 | .46727 | 47.5128 | 51.5338 | 48.67 | 50.28 |  |
|  | 48.00 | 3 | 51.8233 | 1.05434 | .60872 | 49.2042 | 54.4425 | 51.08 | 53.03 |  |
|  | 72.00 | 3 | 53.1400 | 1.97881 | 1.14247 | 48.2244 | 58.0556 | 51.87 | 55.42 |  |
|  | 96.00 | 3 | 52.9100 | 1.38286 | .79839 | 49.4748 | 56.3452 | 51.62 | 54.37 |  |
|  | 120.00 | 3 | 52.7667 | 1.06265 | .61352 | 50.1269 | 55.4064 | 51.75 | 53.87 |  |
|  | Total | 15 | 52.0327 | 1.77429 | .45812 | 51.0501 | 53.0152 | 48.67 | 55.42 |  |
| C1 | 24.00 | 3 | 49.9533 | 1.71419 | .98969 | 45.6951 | 54.2116 | 48.03 | 51.32 |  |
|  | 48.00 | 3 | 53.7367 | .29738 | .17169 | 52.9979 | 54.4754 | 53.42 | 54.01 |  |
|  | 72.00 | 3 | 61.3267 | .82403 | .47576 | 59.2797 | 63.3737 | 60.42 | 62.03 |  |
|  | 96.00 | 3 | 64.1867 | .71710 | .41402 | 62.4053 | 65.9680 | 63.44 | 64.87 |  |
|  | 120.00 | 3 | 45.6267 | .99420 | .57400 | 43.1569 | 48.0964 | 44.86 | 46.75 |  |
|  | Total | 15 | 54.9660 | 7.21327 | 1.86246 | 50.9714 | 58.9606 | 44.86 | 64.87 |  |
| C2 | 24.00 | 3 | 63.7233 | .57709 | .33318 | 62.2898 | 65.1569 | 63.12 | 64.27 |  |
|  | 48.00 | 3 | 64.3000 | .44911 | .25929 | 63.1843 | 65.4157 | 63.89 | 64.78 |  |
|  | 72.00 | 3 | 64.1033 | .61712 | .35629 | 62.5703 | 65.6363 | 63.42 | 64.62 |  |
|  | 96.00 | 3 | 51.1300 | .45574 | .26312 | 49.9979 | 52.2621 | 50.61 | 51.46 |  |
|  | 120.00 | 3 | 33.7333 | .74312 | .42904 | 31.8873 | 35.5794 | 33.15 | 34.57 |  |
|  | Total | 15 | 55.3980 | 12.3609 | 3.19157 | 48.5528 | 62.2432 | 33.15 | 64.78 |  |
| C3 | 24.00 | 3 | 67.7700 | .58026 | .33501 | 66.3286 | 69.2114 | 67.34 | 68.43 |  |
|  | 48.00 | 3 | 59.4200 | .69505 | .40129 | 57.6934 | 61.1466 | 58.73 | 60.12 |  |
|  | 72.00 | 3 | 44.4300 | .81615 | .47120 | 42.4026 | 46.4574 | 43.64 | 45.27 |  |
|  | 96.00 | 3 | 39.4867 | .83524 | .48223 | 37.4118 | 41.5615 | 38.64 | 40.31 |  |
|  | 120.00 | 3 | 19.8967 | .48014 | .27721 | 18.7039 | 21.0894 | 19.37 | 20.31 |  |
|  | Total | 15 | 46.2007 | 17.2070 | 4.4428 | 36.6718 | 55.7296 | 19.37 | 68.43 |  |

| **One-way ANOVA** | | | | | | |
| --- | --- | --- | --- | --- | --- | --- |
|  | | Quadratic sum | df | Mean square | F | Significance |
| CK | Intergroup | 26.626 | 4 | 6.656 | 3.815 | .039 |
|  | Intragroup | 17.448 | 10 | 1.745 |  |  |
|  | Total | 44.073 | 14 |  |  |  |
| C1 | Intergroup | 718.020 | 4 | 179.505 | 172.317 | .000 |
|  | Intragroup | 10.417 | 10 | 1.042 |  |  |
|  | Total | 728.437 | 14 |  |  |  |
| C2 | Intergroup | 2135.740 | 4 | 533.935 | 1593.360 | .000 |
|  | Intragroup | 3.351 | 10 | .335 |  |  |
|  | Total | 2139.091 | 14 |  |  |  |
| C3 | Intergroup | 4140.301 | 4 | 1035.075 | 2143.842 | .000 |
|  | Intragroup | 4.828 | 10 | .483 |  |  |
|  | Total | 4145.129 | 14 |  |  |  |

**Similar subset after Duncan's repeat comparison**

| **CK** | | | |
| --- | --- | --- | --- |
| Duncan^a^ | | | |
| Time | N | A subset of alpha = 0.05 | |
|  |  | 1 | 2 |
| 24.00 | 3 | 49.5233 |  |
| 48.00 | 3 | 51.8233 | 51.8233 |
| 120.00 | 3 |  | 52.7667 |
| 96.00 | 3 |  | 52.9100 |
| 72.00 | 3 |  | 53.1400 |
| Significance |  | .059 | .281 |

| Display the group mean in the same subset of the table | | | | | | |  | | | |
| --- | --- | --- | --- | --- | --- | --- | --- | --- | --- | --- |
| a. Harmonic mean sample size is used = 3.000 | | | | | | |  | | | |
| **C1** | | | | | | | | | | |
| Duncan^a^ | | | | | | | | | | |
| Time | N | A subset of alpha = 0.05 | | | | | | | | |
|  |  | 1 | | 2 | | 3 | | 4 | | 5 |
| 120.00 | 3 | 45.6267 | |  | |  | |  | |  |
| 24.00 | 3 |  | | 49.9533 | |  | |  | |  |
| 48.00 | 3 |  | |  | | 53.7367 | |  | |  |
| 72.00 | 3 |  | |  | |  | | 61.3267 | |  |
| 96.00 | 3 |  | |  | |  | |  | | 64.1867 |
| Significance |  | 1.000 | | 1.000 | | 1.000 | | 1.000 | | 1.000 |
| **C2** | | | | | | | | |  | |
| Duncan^a^ | | | | | | | | |  | |
| Time | N | | A subset of alpha = 0.05 | | | | | |  | |
|  |  |  | 1 | | 2 | | 3 | |  | |
| 120.00 | 3 | | 33.7333 | |  | |  | |  | |
| 96.00 | 3 | |  | | 51.1300 | |  | |  | |
| 24.00 | 3 | |  | |  | | 63.7233 | |  | |
| 72.00 | 3 | |  | |  | | 64.1033 | |  | |
| 48.00 | 3 | |  | |  | | 64.3000 | |  | |
| Significance |  | | 1.000 | | 1.000 | | .271 | |  | |

| **C3** | | | | | | |
| --- | --- | --- | --- | --- | --- | --- |
| Duncan^a^ | | | | | | |
| Time | N | A subset of alpha = 0.05 | | | | |
|  |  | 1 | 2 | 3 | 4 | 5 |
| 120.00 | 3 | 19.8967 |  |  |  |  |
| 96.00 | 3 |  | 39.4867 |  |  |  |
| 72.00 | 3 |  |  | 44.4300 |  |  |
| 48.00 | 3 |  |  |  | 59.4200 |  |
| 24.00 | 3 |  |  |  |  | 67.7700 |
| Significance |  | 1.000 | 1.000 | 1.000 | 1.000 | 1.000 |

| Display the group mean in the same subset of the table |
| --- |
| a. Harmonic mean sample size is used = 3.000 |
