# Supplementary material for: Molluscicidal activity and physiological toxicity of quaternary benzo[c]phenanthridine alkaloids (QBAs) from Macleaya cordata fruits on Oncomelania hupensis
Source: PLoS Negl Trop Dis. 2019 Oct 11;13(10):e0007740. doi: 10.1371/journal.pntd.0007740 (PMC6808491; doi:10.1371/journal.pntd.0007740)
Supplement: S2 Fig — One-way analysis of variance (ANOVA) and simple sequence repeat (SSR, Duncan's repeat comparison of carboxylesterase (CarE) data. (DOC) [file pntd.0007740.s003.doc]

**S3 fig. 2**

One-way analysis of variance (ANOVA) and simple sequence repeat (SSR, Duncan's repeat comparison of **carboxylesterase (CarE) data**

**1.ONEWAY h24 h48 h72 h96 h120 BY C /STATISTICS DESCRIPTIVES /MISSING ANALYSIS /POSTHOC=DUNCAN ALPHA(0.05).**

Numbers of “1.00, 2.00, 3.00, 4.00” in concentration column of the tables mean the concentration of “Control, 1/4LC_50_, 1/2LC_50_ and LC_50_”, respectively.

| **描述** | | | | | | | | |  |
| --- | --- | --- | --- | --- | --- | --- | --- | --- | --- |
| Time(h)/Concentrations | | N | Mean Value | Standard deviation | Standard Error | 95% confidence interval for the mean | | Minimum | Maximum |
|  |  |  |  |  |  | Lower limit | Upper limit |  |  |
| h24 | 1.00 | 3 | .9167 | .03055 | .01764 | .8408 | .9926 | .89 | .95 |
|  | 2.00 | 3 | 1.0533 | .02517 | .01453 | .9908 | 1.1158 | 1.03 | 1.08 |
|  | 3.00 | 3 | 1.2733 | .04041 | .02333 | 1.1729 | 1.3737 | 1.23 | 1.31 |
|  | 4.00 | 3 | 1.1500 | .03000 | .01732 | 1.0755 | 1.2245 | 1.12 | 1.18 |
|  | Total | 12 | 1.0983 | .13921 | .04019 | 1.0099 | 1.1868 | .89 | 1.31 |
| h48 | 1.00 | 3 | .9800 | .03000 | .01732 | .9055 | 1.0545 | .95 | 1.01 |
|  | 2.00 | 3 | 1.2633 | .05132 | .02963 | 1.1359 | 1.3908 | 1.22 | 1.32 |
|  | 3.00 | 3 | .9800 | .05292 | .03055 | .8486 | 1.1114 | .94 | 1.04 |
|  | 4.00 | 3 | .9333 | .01528 | .00882 | .8954 | .9713 | .92 | .95 |
|  | Total | 12 | 1.0392 | .14094 | .04068 | .9496 | 1.1287 | .92 | 1.32 |
| h72 | 1.00 | 3 | .9700 | .03606 | .02082 | .8804 | 1.0596 | .94 | 1.01 |
|  | 2.00 | 3 | .9700 | .04583 | .02646 | .8562 | 1.0838 | .93 | 1.02 |
|  | 3.00 | 3 | .9367 | .03055 | .01764 | .8608 | 1.0126 | .91 | .97 |
|  | 4.00 | 3 | .8733 | .02517 | .01453 | .8108 | .9358 | .85 | .90 |
|  | Total | 12 | .9375 | .05101 | .01473 | .9051 | .9699 | .85 | 1.02 |
| h96 | 1.00 | 3 | .9800 | .03606 | .02082 | .8904 | 1.0696 | .95 | 1.02 |
|  | 2.00 | 3 | .9100 | .03000 | .01732 | .8355 | .9845 | .88 | .94 |
|  | 3.00 | 3 | .9367 | .04509 | .02603 | .8247 | 1.0487 | .89 | .98 |
|  | 4.00 | 3 | .7647 | .03722 | .02149 | .6722 | .8571 | .73 | .80 |
|  | Total | 12 | .8978 | .09028 | .02606 | .8405 | .9552 | .73 | 1.02 |
| h120 | 1.00 | 3 | .9733 | .04509 | .02603 | .8613 | 1.0853 | .93 | 1.02 |
|  | 2.00 | 3 | .8133 | .02517 | .01453 | .7508 | .8758 | .79 | .84 |
|  | 3.00 | 3 | .4733 | .05033 | .02906 | .3483 | .5984 | .42 | .52 |
|  | 4.00 | 3 | .2133 | .05508 | .03180 | .0765 | .3501 | .16 | .27 |
|  | Total | 12 | .6183 | .31098 | .08977 | .4207 | .8159 | .16 | 1.02 |

| **One-way ANOVA** | | | | | | |
| --- | --- | --- | --- | --- | --- | --- |
|  | | Quadratic sum | df | Mean square | F | Significance |
| h24 | Intergroup | .205 | 3 | .068 | 66.656 | .000 |
|  | Intragroup | .008 | 8 | .001 |  |  |
|  | Total | .213 | 11 |  |  |  |
| h48 | Intergroup | .205 | 3 | .068 | 41.697 | .000 |
|  | Intragroup | .013 | 8 | .002 |  |  |
|  | Total | .218 | 11 |  |  |  |
| h72 | Intergroup | .019 | 3 | .006 | 5.018 | .030 |
|  | Intragroup | .010 | 8 | .001 |  |  |
|  | Total | .029 | 11 |  |  |  |
| h96 | Intergroup | .078 | 3 | .026 | 18.610 | .001 |
|  | Intragroup | .011 | 8 | .001 |  |  |
|  | Total | .090 | 11 |  |  |  |
| h120 | Intergroup | 1.047 | 3 | .349 | 169.603 | .000 |
|  | Intragroup | .016 | 8 | .002 |  |  |
|  | Total | 1.064 | 11 |  |  |  |

**Similar subset after Duncan's repeat comparison**

| **h24** | | | | | | | | |
| --- | --- | --- | --- | --- | --- | --- | --- | --- |
| Duncan^a^ | | | | | | | | |
| C | N | A subset of alpha = 0.05 | | | | | | |
|  |  | 1 | | 2 | | 3 | | 4 |
| 1.00 | 3 | .9167 | |  | |  | |  |
| 2.00 | 3 |  | | 1.0533 | |  | |  |
| 4.00 | 3 |  | |  | | 1.1500 | |  |
| 3.00 | 3 |  | |  | |  | | 1.2733 |
| Significance |  | 1.000 | | 1.000 | | 1.000 | | 1.000 |
| Display the group mean in the same subset of the table | | | | | | | | |
| a. Harmonic mean sample size is used = 3.000 | | | | | | | | |
| **h48** | | | | | | |  | |
| Duncan^a^ | | | | | | |  | |
| C | N | | A subset of alpha = 0.05 | | | |  | |
|  |  |  | 1 | | 2 | |  | |
| 4.00 | 3 | | .9333 | |  | |  | |
| 1.00 | 3 | | .9800 | |  | |  | |
| 3.00 | 3 | | .9800 | |  | |  | |
| 2.00 | 3 | |  | | 1.2633 | |  | |
| Significance |  | | .213 | | 1.000 | |  | |
| **h72** | | | | | | |  | |
| Duncan^a^ | | | | | | |  | |
| C | N | | A subset of alpha = 0.05 | | | |  | |
|  |  |  | 1 | | 2 | |  | |
| 4.00 | 3 | | .8733 | |  | |  | |
| 3.00 | 3 | | .9367 | | .9367 | |  | |
| 1.00 | 3 | |  | | .9700 | |  | |
| 2.00 | 3 | |  | | .9700 | |  | |
| Significance |  | | .059 | | .299 | |  | |
| **h96** | | | | | | |  | |
| Duncan^a^ | | | | | | |  | |
| C | N | | A subset of alpha = 0.05 | | | |  | |
|  |  |  | 1 | | 2 | |  | |
| 4.00 | 3 | | .7647 | |  | |  | |
| 2.00 | 3 | |  | | .9100 | |  | |
| 3.00 | 3 | |  | | .9367 | |  | |
| 1.00 | 3 | |  | | .9800 | |  | |
| Significance |  | | 1.000 | | .059 | |  | |
| **h120** | | | | | | | | |
| Duncan^a^ | | | | | | | | |
| C | N | A subset of alpha = 0.05 | | | | | | |
|  |  | 1 | | 2 | | 3 | | 4 |
| 4.00 | 3 | .2133 | |  | |  | |  |
| 3.00 | 3 |  | | .4733 | |  | |  |
| 2.00 | 3 |  | |  | | .8133 | |  |
| 1.00 | 3 |  | |  | |  | | .9733 |
| Significance |  | 1.000 | | 1.000 | | 1.000 | | 1.000 |

| Display the group mean in the same subset of the table |
| --- |
| a. Harmonic mean sample size is used = 3.000 |

**2.ONEWAY CK C1 C2 C3 BY Time /STATISTICS DESCRIPTIVES /MISSING ANALYSIS /POSTHOC=DUNCAN ALPHA(0.05).**

|  | | | | | | | | | | | | | | |  |
| --- | --- | --- | --- | --- | --- | --- | --- | --- | --- | --- | --- | --- | --- | --- | --- |
| Concentrations/ Time(h) | | N | | Mean Value | | Standard deviation | | Standard Error | | 95% confidence interval for the mean | | | | Minimum | Maximum |
|  |  |  |  |  |  |  |  |  |  | Lower limit | | Upper limit | |  |  |
| CK | 24.00 | 3 | | .9167 | | .03055 | | .01764 | | .8408 | | .9926 | | .89 | .95 |
|  | 48.00 | 3 | | .9800 | | .03000 | | .01732 | | .9055 | | 1.0545 | | .95 | 1.01 |
|  | 72.00 | 3 | | .9700 | | .03606 | | .02082 | | .8804 | | 1.0596 | | .94 | 1.01 |
|  | 96.00 | 3 | | .9800 | | .03606 | | .02082 | | .8904 | | 1.0696 | | .95 | 1.02 |
|  | 120.00 | 3 | | .9733 | | .04509 | | .02603 | | .8613 | | 1.0853 | | .93 | 1.02 |
|  | Total | 15 | | .9640 | | .03924 | | .01013 | | .9423 | | .9857 | | .89 | 1.02 |
| C1 | 24.00 | 3 | | 1.0533 | | .02517 | | .01453 | | .9908 | | 1.1158 | | 1.03 | 1.08 |
|  | 48.00 | 3 | | 1.2633 | | .05132 | | .02963 | | 1.1359 | | 1.3908 | | 1.22 | 1.32 |
|  | 72.00 | 3 | | .9700 | | .04583 | | .02646 | | .8562 | | 1.0838 | | .93 | 1.02 |
|  | 96.00 | 3 | | .9100 | | .03000 | | .01732 | | .8355 | | .9845 | | .88 | .94 |
|  | 120.00 | 3 | | .8133 | | .02517 | | .01453 | | .7508 | | .8758 | | .79 | .84 |
|  | Total | 15 | | 1.0020 | | .16077 | | .04151 | | .9130 | | 1.0910 | | .79 | 1.32 |
| C2 | 24.00 | 3 | | 1.2733 | | .04041 | | .02333 | | 1.1729 | | 1.3737 | | 1.23 | 1.31 |
|  | 48.00 | 3 | | .9800 | | .05292 | | .03055 | | .8486 | | 1.1114 | | .94 | 1.04 |
|  | 72.00 | 3 | | .9367 | | .03055 | | .01764 | | .8608 | | 1.0126 | | .91 | .97 |
|  | 96.00 | 3 | | .9367 | | .04509 | | .02603 | | .8247 | | 1.0487 | | .89 | .98 |
|  | 120.00 | 3 | | .4733 | | .05033 | | .02906 | | .3483 | | .5984 | | .42 | .52 |
|  | Total | 15 | | .9200 | | .26798 | | .06919 | | .7716 | | 1.0684 | | .42 | 1.31 |
| C3 | 24.00 | 3 | | 1.1500 | | .03000 | | .01732 | | 1.0755 | | 1.2245 | | 1.12 | 1.18 |
|  | 48.00 | 3 | | .9333 | | .01528 | | .00882 | | .8954 | | .9713 | | .92 | .95 |
|  | 72.00 | 3 | | .8733 | | .02517 | | .01453 | | .8108 | | .9358 | | .85 | .90 |
|  | 96.00 | 3 | | .7647 | | .03722 | | .02149 | | .6722 | | .8571 | | .73 | .80 |
|  | 120.00 | 3 | | .2133 | | .05508 | | .03180 | | .0765 | | .3501 | | .16 | .27 |
|  | Total | 15 | | .7869 | | .32547 | | .08404 | | .6067 | | .9672 | | .16 | 1.18 |
| **One-way ANOVA** | | | | | | | | | | | | |  | | |
|  | | | Quadratic sum | | df | | Mean square | | F | | Significance | |  | | |
| CK | Intergroup | | .009 | | 4 | | .002 | | 1.668 | | .233 | |  | | |
|  | Intragroup | | .013 | | 10 | | .001 | |  | |  | |  | | |
|  | Total | | .022 | | 14 | |  | |  | |  | |  | | |
| C1 | Intergroup | | .348 | | 4 | | .087 | | 63.051 | | .000 | |  | | |
|  | Intragroup | | .014 | | 10 | | .001 | |  | |  | |  | | |
|  | Total | | .362 | | 14 | |  | |  | |  | |  | | |
| C2 | Intergroup | | .986 | | 4 | | .246 | | 124.018 | | .000 | |  | | |
|  | Intragroup | | .020 | | 10 | | .002 | |  | |  | |  | | |
|  | Total | | 1.005 | | 14 | |  | |  | |  | |  | | |
| C3 | Intergroup | | 1.471 | | 4 | | .368 | | 297.212 | | .000 | |  | | |
|  | Intragroup | | .012 | | 10 | | .001 | |  | |  | |  | | |
|  | Total | | 1.483 | | 14 | |  | |  | |  | |  | | |

**Similar subset after Duncan's repeat comparison**

| **CK** | | | | | |  | |
| --- | --- | --- | --- | --- | --- | --- | --- |
| Duncan^a^ | | | | | |  | |
| Time | N | | A subset of alpha = 0.05 | | |  | |
|  |  |  | 1 | | |  | |
| 24.00 | 3 | | .9167 | | |  | |
| 72.00 | 3 | | .9700 | | |  | |
| 120.00 | 3 | | .9733 | | |  | |
| 48.00 | 3 | | .9800 | | |  | |
| 96.00 | 3 | | .9800 | | |  | |
| Significance |  | | .076 | | |  | |
| Display the group mean in the same subset of the table | | | | | |  | |
| a. Harmonic mean sample size is used = 3.000 | | | | | |  | |
| **C1** | | | | | | | |
| Duncan^a^ | | | | | | | |
| Time | N | A subset of alpha = 0.05 | | | | | |
|  |  | 1 | | 2 | 3 | | 4 |
| 120.00 | 3 | .8133 | |  |  | |  |
| 96.00 | 3 |  | | .9100 |  | |  |
| 72.00 | 3 |  | | .9700 |  | |  |
| 24.00 | 3 |  | |  | 1.0533 | |  |
| 48.00 | 3 |  | |  |  | | 1.2633 |
| Significance |  | 1.000 | | .076 | 1.000 | | 1.000 |
| **C2** | | | | | | |  |
| Duncan^a^ | | | | | | |  |
| Time | N | A subset of alpha = 0.05 | | | | |  |
|  |  | 1 | | 2 | 3 | |  |
| 120.00 | 3 | .4733 | |  |  | |  |
| 72.00 | 3 |  | | .9367 |  | |  |
| 96.00 | 3 |  | | .9367 |  | |  |
| 48.00 | 3 |  | | .9800 |  | |  |
| 24.00 | 3 |  | |  | 1.2733 | |  |
| Significance |  | 1.000 | | .282 | 1.000 | |  |
| **C3** | | | | | | | |
| Duncan^a^ | | | | | | | |
| Time | N | A subset of alpha = 0.05 | | | | | |
|  |  | 1 | | 2 | 3 | | 4 |
| 120.00 | 3 | .2133 | |  |  | |  |
| 96.00 | 3 |  | | .7647 |  | |  |
| 72.00 | 3 |  | |  | .8733 | |  |
| 48.00 | 3 |  | |  | .9333 | |  |
| 24.00 | 3 |  | |  |  | | 1.1500 |
| Significance |  | 1.000 | | 1.000 | .063 | | 1.000 |
| Display the group mean in the same subset of the table | | | | | | | |
| a. Harmonic mean sample size is used = 3.000 | | | | | | | |
