# Supplementary material for: Molluscicidal activity and physiological toxicity of quaternary benzo[c]phenanthridine alkaloids (QBAs) from Macleaya cordata fruits on Oncomelania hupensis
Source: PLoS Negl Trop Dis. 2019 Oct 11;13(10):e0007740. doi: 10.1371/journal.pntd.0007740 (PMC6808491; doi:10.1371/journal.pntd.0007740)
Supplement: S3 Fig — One-way analysis of variance (ANOVA) and simple sequence repeat (SSR, Duncan's repeat comparison of acid phosphatase (ACP) data (Fig 3A). Effects of quaternary benzo[c]phenanthridine alkaloids from Macleaya cordata fruits on alkaline phosphatase (AKP; B) activity in the liver of Oncomelania hupensis snails. One-way analysis of variance (ANOVA) and simple sequence repeat (SSR, Duncan's repeat comparison of alkaline phosphatase (AKP) data(Fig 3B). (DOC) [file pntd.0007740.s004.doc]

**S4 fig. 3A**

One-way analysis of variance (ANOVA) and simple sequence repeat (SSR, Duncan's repeat comparison of **acid phosphatase (ACP) data**

1. **ONEWAY h24 h48 h72 h96 h120 BY Concentrations (C) /STATISTICS DESCRIPTIVES /MISSING ANALYSIS /POSTHOC=DUNCAN ALPHA(0.05)**.

Numbers of “1.00, 2.00, 3.00, 4.00” in concentration column of the tables mean the concentration of “Control, 1/4LC_50_, 1/2LC_50_ and LC_50_”, respectively.

| STATISTICS DESCRIPTIVES | | | | | | | | | | | | | | |  |
| --- | --- | --- | --- | --- | --- | --- | --- | --- | --- | --- | --- | --- | --- | --- | --- |
| Time(h)/Concentrations | | N | Mean Value | | Standard deviation | | Standard Error | | | 95% confidence interval for the mean | | | | Minimum | Maximum |
|  |  |  |  |  |  |  |  |  |  | Lower limit | | Upper limit | |  |  |
| h24 | 1.00 | 3 | 3.3233 | | .09609 | | .05548 | | | 3.0846 | | 3.5620 | | 3.22 | 3.41 |
|  | 2.00 | 3 | 5.0100 | | .06557 | | .03786 | | | 4.8471 | | 5.1729 | | 4.94 | 5.07 |
|  | 3.00 | 3 | 4.1500 | | .10817 | | .06245 | | | 3.8813 | | 4.4187 | | 4.06 | 4.27 |
|  | 4.00 | 3 | 3.8200 | | .17349 | | .10017 | | | 3.3890 | | 4.2510 | | 3.67 | 4.01 |
|  | Total | 12 | 4.0758 | | .64950 | | .18749 | | | 3.6632 | | 4.4885 | | 3.22 | 5.07 |
| h48 | 1.00 | 3 | 3.2367 | | .11676 | | .06741 | | | 2.9466 | | 3.5267 | | 3.11 | 3.34 |
|  | 2.00 | 3 | 4.3933 | | .12503 | | .07219 | | | 4.0827 | | 4.7039 | | 4.27 | 4.52 |
|  | 3.00 | 3 | 3.0500 | | .07000 | | .04041 | | | 2.8761 | | 3.2239 | | 2.98 | 3.12 |
|  | 4.00 | 3 | 2.7267 | | .13317 | | .07688 | | | 2.3959 | | 3.0575 | | 2.58 | 2.84 |
|  | Total | 12 | 3.3517 | | .66357 | | .19156 | | | 2.9301 | | 3.7733 | | 2.58 | 4.52 |
| h72 | 1.00 | 3 | 3.2500 | | .11790 | | .06807 | | | 2.9571 | | 3.5429 | | 3.15 | 3.38 |
|  | 2.00 | 3 | 2.7933 | | .04163 | | .02404 | | | 2.6899 | | 2.8968 | | 2.76 | 2.84 |
|  | 3.00 | 3 | 2.4200 | | .08544 | | .04933 | | | 2.2078 | | 2.6322 | | 2.34 | 2.51 |
|  | 4.00 | 3 | 2.1700 | | .04583 | | .02646 | | | 2.0562 | | 2.2838 | | 2.12 | 2.21 |
|  | Total | 12 | 2.6583 | | .43073 | | .12434 | | | 2.3847 | | 2.9320 | | 2.12 | 3.38 |
| h96 | 1.00 | 3 | 3.1133 | | .10017 | | .05783 | | | 2.8645 | | 3.3622 | | 3.01 | 3.21 |
|  | 2.00 | 3 | 2.3433 | | .07024 | | .04055 | | | 2.1689 | | 2.5178 | | 2.27 | 2.41 |
|  | 3.00 | 3 | 2.3767 | | .03512 | | .02028 | | | 2.2894 | | 2.4639 | | 2.34 | 2.41 |
|  | 4.00 | 3 | 1.7233 | | .05508 | | .03180 | | | 1.5865 | | 1.8601 | | 1.67 | 1.78 |
|  | Total | 12 | 2.3892 | | .51773 | | .14946 | | | 2.0602 | | 2.7181 | | 1.67 | 3.21 |
| h120 | 1.00 | 3 | 3.1233 | | .13614 | | .07860 | | | 2.7851 | | 3.4615 | | 2.97 | 3.23 |
|  | 2.00 | 3 | 1.6833 | | .16010 | | .09244 | | | 1.2856 | | 2.0811 | | 1.52 | 1.84 |
|  | 3.00 | 3 | 1.1867 | | .06028 | | .03480 | | | 1.0369 | | 1.3364 | | 1.13 | 1.25 |
|  | 4.00 | 3 | .9800 | | .06557 | | .03786 | | | .8171 | | 1.1429 | | .92 | 1.05 |
|  | Total | 12 | 1.7433 | | .87935 | | .25385 | | | 1.1846 | | 2.3020 | | .92 | 3.23 |
| **One-way ANOVA** | | | | | | | | | | | | |  | | |
|  | | Quadratic sum | | df | | Mean square | | | F | | Significance | |  | | |
| h24 | Intergroup | 4.530 | | 3 | | 1.510 | | | 109.148 | | .000 | |  | | |
|  | Intragroup | .111 | | 8 | | .014 | | |  | |  | |  | | |
|  | Total | 4.640 | | 11 | |  | | |  | |  | |  | | |
| h48 | Intergroup | 4.740 | | 3 | | 1.580 | | | 121.767 | | .000 | |  | | |
|  | Intragroup | .104 | | 8 | | .013 | | |  | |  | |  | | |
|  | Total | 4.844 | | 11 | |  | |  | | |  | |  | | |
| h72 | Intergroup | 1.991 | | 3 | | .664 | | 106.029 | | | .000 | |  | | |
|  | Intragroup | .050 | | 8 | | .006 | |  | | |  | |  | | |
|  | Total | 2.041 | | 11 | |  | |  | | |  | |  | | |
| h96 | Intergroup | 2.910 | | 3 | | .970 | | 201.735 | | | .000 | |  | | |
|  | Intragroup | .038 | | 8 | | .005 | |  | | |  | |  | | |
|  | Total | 2.948 | | 11 | |  | |  | | |  | |  | | |
| h120 | Intergroup | 8.402 | | 3 | | 2.801 | | 215.014 | | | .000 | |  | | |
|  | Intragroup | .104 | | 8 | | .013 | |  | | |  | |  | | |
|  | Total | 8.506 | | 11 | |  | |  | | |  | |  | | |

**Similar subset after Duncan's repeat comparison**

| **h24** | | | | | | | | | | |
| --- | --- | --- | --- | --- | --- | --- | --- | --- | --- | --- |
| Duncan^a^ | | | | | | | | | | |
| Concentrations | N | | A subset of alpha = 0.05 | | | | | | | |
|  |  |  | 1 | | 2 | | 3 | | 4 | |
| 1.00 | 3 | | 3.3233 | |  | |  | |  | |
| 4.00 | 3 | |  | | 3.8200 | |  | |  | |
| 3.00 | 3 | |  | |  | | 4.1500 | |  | |
| 2.00 | 3 | |  | |  | |  | | 5.0100 | |
| Significance |  | | 1.000 | | 1.000 | | 1.000 | | 1.000 | |
| Display the group mean in the same subset of the table | | | | | | | | | | |
| a. Harmonic mean sample size is used = 3.000 | | | | | | | | | | |
| **h48** | | | | | | | | | |  |
| Duncan^a^ | | | | | | | | | |  |
| Concentrations | N | | | A subset of alpha = 0.05 | | | | | |  |
|  |  |  |  | 1 | | 2 | | 3 | |  |
| 4.00 | 3 | | | 2.7267 | |  | |  | |  |
| 3.00 | 3 | | |  | | 3.0500 | |  | |  |
| 1.00 | 3 | | |  | | 3.2367 | |  | |  |
| 2.00 | 3 | | |  | |  | | 4.3933 | |  |
| Significance |  | | | 1.000 | | .080 | | 1.000 | |  |
| **h72** | | | | | | | | | | |
| Duncan^a^ | | | | | | | | | | |
| Concentrations | | N | A subset of alpha = 0.05 | | | | | | | |
|  |  |  | 1 | | 2 | | 3 | | 4 | |
| 4.00 | | 3 | 2.1700 | |  | |  | |  | |
| 3.00 | | 3 |  | | 2.4200 | |  | |  | |
| 2.00 | | 3 |  | |  | | 2.7933 | |  | |
| 1.00 | | 3 |  | |  | |  | | 3.2500 | |
| Significance | |  | 1.000 | | 1.000 | | 1.000 | | 1.000 | |
| **h96** | | | | | | | | | |  |
| Duncan^a^ | | | | | | | | | |  |
| Concentrations | | N | | A subset of alpha = 0.05 | | | | | |  |
|  |  |  |  | 1 | | 2 | | 3 | |  |
| 4.00 | | 3 | | 1.7233 | |  | |  | |  |
| 2.00 | | 3 | |  | | 2.3433 | |  | |  |
| 3.00 | | 3 | |  | | 2.3767 | |  | |  |
| 1.00 | | 3 | |  | |  | | 3.1133 | |  |
| Significance | |  | | 1.000 | | .572 | | 1.000 | |  |
| **h120** | | | | | | | | | |  |
| Duncan^a^ | | | | | | | | | |  |
| Concentrations | | N | | A subset of alpha = 0.05 | | | | | |  |
|  |  |  |  | 1 | | 2 | | 3 | |  |
| 4.00 | | 3 | | .9800 | |  | |  | |  |
| 3.00 | | 3 | | 1.1867 | |  | |  | |  |
| 2.00 | | 3 | |  | | 1.6833 | |  | |  |
| 1.00 | | 3 | |  | |  | | 3.1233 | |  |
| Significance | |  | | .057 | | 1.000 | | 1.000 | |  |
| Display the group mean in the same subset of the table | | | | | | | | | |  |
| a. Harmonic mean sample size is used = 3.000 | | | | | | | | | |  |

1. **ONEWAY CK C1 C2 C3 BY Time /STATISTICS DESCRIPTIVES /MISSING ANALYSIS /POSTHOC=DUNCAN ALPHA (0.05).**

| STATISTICS DESCRIPTIVES | | | | | | | | |  |
| --- | --- | --- | --- | --- | --- | --- | --- | --- | --- |
| Concentrations/Time(h) | | N | Mean Value | Standard deviation | Standard Error | 95% confidence interval for the mean | | Minimum | Maximum |
|  |  |  |  |  |  | Lower limit | Upper limit |  |  |
| CK | 24.00 | 3 | 3.3233 | .09609 | .05548 | 3.0846 | 3.5620 | 3.22 | 3.41 |
|  | 48.00 | 3 | 3.2367 | .11676 | .06741 | 2.9466 | 3.5267 | 3.11 | 3.34 |
|  | 72.00 | 3 | 3.2500 | .11790 | .06807 | 2.9571 | 3.5429 | 3.15 | 3.38 |
|  | 96.00 | 3 | 3.1133 | .10017 | .05783 | 2.8645 | 3.3622 | 3.01 | 3.21 |
|  | 120.00 | 3 | 3.1233 | .13614 | .07860 | 2.7851 | 3.4615 | 2.97 | 3.23 |
|  | Total | 15 | 3.2093 | .12725 | .03286 | 3.1389 | 3.2798 | 2.97 | 3.41 |
| C1 | 24.00 | 3 | 5.0100 | .06557 | .03786 | 4.8471 | 5.1729 | 4.94 | 5.07 |
|  | 48.00 | 3 | 4.3933 | .12503 | .07219 | 4.0827 | 4.7039 | 4.27 | 4.52 |
|  | 72.00 | 3 | 2.7933 | .04163 | .02404 | 2.6899 | 2.8968 | 2.76 | 2.84 |
|  | 96.00 | 3 | 2.3433 | .07024 | .04055 | 2.1689 | 2.5178 | 2.27 | 2.41 |
|  | 120.00 | 3 | 1.6833 | .16010 | .09244 | 1.2856 | 2.0811 | 1.52 | 1.84 |
|  | Total | 15 | 3.2447 | 1.30312 | .33646 | 2.5230 | 3.9663 | 1.52 | 5.07 |
| C2 | 24.00 | 3 | 4.1500 | .10817 | .06245 | 3.8813 | 4.4187 | 4.06 | 4.27 |
|  | 48.00 | 3 | 3.0500 | .07000 | .04041 | 2.8761 | 3.2239 | 2.98 | 3.12 |
|  | 72.00 | 3 | 2.4200 | .08544 | .04933 | 2.2078 | 2.6322 | 2.34 | 2.51 |
|  | 96.00 | 3 | 2.3767 | .03512 | .02028 | 2.2894 | 2.4639 | 2.34 | 2.41 |
|  | 120.00 | 3 | 1.1867 | .06028 | .03480 | 1.0369 | 1.3364 | 1.13 | 1.25 |
|  | Total | 15 | 2.6367 | 1.00327 | .25904 | 2.0811 | 3.1923 | 1.13 | 4.27 |
| C3 | 24.00 | 3 | 3.8200 | .17349 | .10017 | 3.3890 | 4.2510 | 3.67 | 4.01 |
|  | 48.00 | 3 | 2.7267 | .13317 | .07688 | 2.3959 | 3.0575 | 2.58 | 2.84 |
|  | 72.00 | 3 | 2.1700 | .04583 | .02646 | 2.0562 | 2.2838 | 2.12 | 2.21 |
|  | 96.00 | 3 | 1.7233 | .05508 | .03180 | 1.5865 | 1.8601 | 1.67 | 1.78 |
|  | 120.00 | 3 | .9800 | .06557 | .03786 | .8171 | 1.1429 | .92 | 1.05 |
|  | Total | 15 | 2.2840 | .99512 | .25694 | 1.7329 | 2.8351 | .92 | 4.01 |

| **One-way ANOVA** | | | | | | |
| --- | --- | --- | --- | --- | --- | --- |
|  | | Quadratic sum | df | Mean square | F | Significance |
| CK | Intergroup | .096 | 4 | .024 | 1.837 | .198 |
|  | Intragroup | .131 | 10 | .013 |  |  |
|  | Total | .227 | 14 |  |  |  |
| C1 | Intergroup | 23.669 | 4 | 5.917 | 566.427 | .000 |
|  | Intragroup | .104 | 10 | .010 |  |  |
|  | Total | 23.774 | 14 |  |  |  |
| C2 | Intergroup | 14.034 | 4 | 3.509 | 609.829 | .000 |
|  | Intragroup | .058 | 10 | .006 |  |  |
|  | Total | 14.092 | 14 |  |  |  |
| C3 | Intergroup | 13.749 | 4 | 3.437 | 300.110 | .000 |
|  | Intragroup | .115 | 10 | .011 |  |  |
|  | Total | 13.864 | 14 |  |  |  |

**Similar subset after Duncan's repeat comparison**

| **CK** | | | | | | |  | | | | | |
| --- | --- | --- | --- | --- | --- | --- | --- | --- | --- | --- | --- | --- |
| Duncan^a^ | | | | | | |  | | | | | |
| Time (h) | N | | A subset of alpha = 0.05 | | | |  | | | | | |
|  |  |  | 1 | | | |  | | | | | |
| 96.00 | 3 | | 3.1133 | | | |  | | | | | |
| 120.00 | 3 | | 3.1233 | | | |  | | | | | |
| 48.00 | 3 | | 3.2367 | | | |  | | | | | |
| 72.00 | 3 | | 3.2500 | | | |  | | | | | |
| 24.00 | 3 | | 3.3233 | | | |  | | | | | |
| Significance |  | | .066 | | | |  | | | | | |
| Display the group mean in the same subset of the table | | | | | | |  | | | | | |
| a. Harmonic mean sample size is used = 3.000 | | | | | | |  | | | | | |
| **C1** | | | | | | | | | | | |  |
| Duncan^a^ | | | | | | | | | | | |  |
| Time (h) | N | A subset of alpha = 0.05 | | | | | | | | | |  |
|  |  | 1 | | 2 | | 3 | | 4 | | 5 | |  |
| 120.00 | 3 | 1.6833 | |  | |  | |  | |  | |  |
| 96.00 | 3 |  | | 2.3433 | |  | |  | |  | |  |
| 72.00 | 3 |  | |  | | 2.7933 | |  | |  | |  |
| 48.00 | 3 |  | |  | |  | | 4.3933 | |  | |  |
| 24.00 | 3 |  | |  | |  | |  | | 5.0100 | |  |
| Significance |  | 1.000 | | 1.000 | | 1.000 | | 1.000 | | 1.000 | |  |
| **C2** | | | | | | |  | | | | | |
| Duncan^a^ | | | | | | |  | | | | | |
| Time (h) | N | A subset of alpha = 0.05 | | | | | | | |  | | |
|  |  | 1 | | 2 | | 3 | | | 4 |  | | |
| 120.00 | 3 | 1.1867 | |  | |  | | |  |  | | |
| 96.00 | 3 |  | | 2.3767 | |  | | |  |  | | |
| 72.00 | 3 |  | | 2.4200 | |  | | |  |  | | |
| 48.00 | 3 |  | |  | | 3.0500 | | |  |  | | |
| 24.00 | 3 |  | |  | |  | | | 4.1500 | |  | |
| Significance |  | 1.000 | | .500 | | 1.000 | | | 1.000 | |  | |
| **C3** | | | | |  | | | | | | | |
| Duncan^a^ | | | | |  | | | | | | | |
| Time (h) | N | A subset of alpha = 0.05 | | | | | | | | |  | |
|  |  | 1 | | 2 | | 3 | | 4 | | | 5 | |
| 120.00 | 3 | .9800 | |  | |  | |  | | |  | |
| 96.00 | 3 |  | | 1.7233 | |  | |  | | |  | |
| 72.00 | 3 |  | |  | | 2.1700 | |  | | |  | |
| 48.00 | 3 |  | |  | |  | | 2.7267 | | |  | |
| 24.00 | 3 |  | |  | |  | |  | | | 3.8200 | |
| Significance |  | 1.000 | | 1.000 | | 1.000 | | 1.000 | | | 1.000 | |

| Display the group mean in the same subset of the table |
| --- |
| a. Harmonic mean sample size is used = 3.000 |

**S5 fig. 3B**

One-way analysis of variance (ANOVA) and simple sequence repeat (SSR, Duncan's repeat comparison of **alkaline phosphatase (AKP) data**

1. **ONEWAY h24 h48 h72 h96 h120 BY Concentrations /STATISTICS DESCRIPTIVES /MISSING ANALYSIS /POSTHOC=DUNCAN ALPHA(0.05).**

Numbers of “1.00, 2.00, 3.00, 4.00” in concentration column of the tables mean the concentration of “Control, 1/4LC_50_, 1/2LC_50_ and LC_50_”, respectively.

| STATISTICS DESCRIPTIVES： |
| --- |

| Time/Concentrations | | N | Mean Value | | Standard deviation | | Standard Error | | 95% confidence interval for the mean | | | | Minimum | Maximum |
| --- | --- | --- | --- | --- | --- | --- | --- | --- | --- | --- | --- | --- | --- | --- |
|  |  |  |  |  |  |  |  |  | Lower limit | | Upper limit | |  |  |
| h24 | 1.00 | 3 | 1.4833 | | .01528 | | .00882 | | 1.4454 | | 1.5213 | | 1.47 | 1.50 |
|  | 2.00 | 3 | 2.1367 | | .01528 | | .00882 | | 2.0987 | | 2.1746 | | 2.12 | 2.15 |
|  | 3.00 | 3 | 1.9800 | | .04583 | | .02646 | | 1.8662 | | 2.0938 | | 1.94 | 2.03 |
|  | 4.00 | 3 | 1.8067 | | .04163 | | .02404 | | 1.7032 | | 1.9101 | | 1.76 | 1.84 |
|  | Total | 12 | 1.8517 | | .25491 | | .07359 | | 1.6897 | | 2.0136 | | 1.47 | 2.15 |
| h48 | 1.00 | 3 | 1.5133 | | .05508 | | .03180 | | 1.3765 | | 1.6501 | | 1.46 | 1.57 |
|  | 2.00 | 3 | 2.1800 | | .02000 | | .01155 | | 2.1303 | | 2.2297 | | 2.16 | 2.20 |
|  | 3.00 | 3 | 1.5067 | | .03512 | | .02028 | | 1.4194 | | 1.5939 | | 1.47 | 1.54 |
|  | 4.00 | 3 | 1.3933 | | .04726 | | .02728 | | 1.2759 | | 1.5107 | | 1.34 | 1.43 |
|  | Total | 12 | 1.6483 | | .32638 | | .09422 | | 1.4410 | | 1.8557 | | 1.34 | 2.20 |
| h72 | 1.00 | 3 | 1.4967 | | .03786 | | .02186 | | 1.4026 | | 1.5907 | | 1.47 | 1.54 |
|  | 2.00 | 3 | 1.3800 | | .03000 | | .01732 | | 1.3055 | | 1.4545 | | 1.35 | 1.41 |
|  | 3.00 | 3 | 1.2067 | | .03512 | | .02028 | | 1.1194 | | 1.2939 | | 1.17 | 1.24 |
|  | 4.00 | 3 | 1.0733 | | .05033 | | .02906 | | .9483 | | 1.1984 | | 1.02 | 1.12 |
|  | Total | 12 | 1.2892 | | .17223 | | .04972 | | 1.1797 | | 1.3986 | | 1.02 | 1.54 |
| h96 | 1.00 | 3 | 1.4667 | | .03055 | | .01764 | | 1.3908 | | 1.5426 | | 1.44 | 1.50 |
|  | 2.00 | 3 | 1.1833 | | .04509 | | .02603 | | 1.0713 | | 1.2953 | | 1.14 | 1.23 |
|  | 3.00 | 3 | 1.1833 | | .03512 | | .02028 | | 1.0961 | | 1.2706 | | 1.15 | 1.22 |
|  | 4.00 | 3 | .8333 | | .02082 | | .01202 | | .7816 | | .8850 | | .81 | .85 |
|  | Total | 12 | 1.1667 | | .23631 | | .06822 | | 1.0165 | | 1.3168 | | .81 | 1.50 |
| h120 | 1.00 | 3 | 1.4467 | | .02517 | | .01453 | | 1.3842 | | 1.5092 | | 1.42 | 1.47 |
|  | 2.00 | 3 | .8233 | | .07638 | | .04410 | | .6336 | | 1.0131 | | .74 | .89 |
|  | 3.00 | 3 | .6667 | | .01528 | | .00882 | | .6287 | | .7046 | | .65 | .68 |
|  | 4.00 | 3 | .5900 | | .05568 | | .03215 | | .4517 | | .7283 | | .54 | .65 |
|  | Total | 12 | .8817 | | .35437 | | .10230 | | .6565 | | 1.1068 | | .54 | 1.47 |
| **One-way ANOVA** | | | | | | | | | | | |  | | |
|  | | Quadratic sum | | df | | Mean square | | F | | Significance | |  | | |
| h24 | Intergroup | .706 | | 3 | | .235 | | 218.966 | | .000 | |  | | |
|  | Intragroup | .009 | | 8 | | .001 | |  | |  | |  | | |
|  | Total | .715 | | 11 | |  | |  | |  | |  | | |
| h48 | Intergroup | 1.158 | | 3 | | .386 | | 223.762 | | .000 | |  | | |
|  | Intragroup | .014 | | 8 | | .002 | |  | |  | |  | | |
|  | Total | 1.172 | | 11 | |  | |  | |  | |  | | |
| h72 | Intergroup | .314 | | 3 | | .105 | | 68.654 | | .000 | |  | | |
|  | Intragroup | .012 | | 8 | | .002 | |  | |  | |  | | |
|  | Total | .326 | | 11 | |  | |  | |  | |  | | |
| h96 | Intergroup | .605 | | 3 | | .202 | | 174.101 | | .000 | |  | | |
|  | Intragroup | .009 | | 8 | | .001 | |  | |  | |  | | |
|  | Total | .614 | | 11 | |  | |  | |  | |  | | |
| h120 | Intergroup | 1.362 | | 3 | | .454 | | 185.274 | | .000 | |  | | |
|  | Intragroup | .020 | | 8 | | .002 | |  | |  | |  | | |
|  | Total | 1.381 | | 11 | |  | |  | |  | |  | | |

**Similar subset after Duncan's repeat comparison**

| **h24** | | | | | | | | | |
| --- | --- | --- | --- | --- | --- | --- | --- | --- | --- |
| Duncan^a^ | | | | | | | | | |
| Concentrations | N | A subset of alpha = 0.05 | | | | | | | |
|  |  | 1 | | 2 | | 3 | | 4 | |
| 1.00 | 3 | 1.4833 | |  | |  | |  | |
| 4.00 | 3 |  | | 1.8067 | |  | |  | |
| 3.00 | 3 |  | |  | | 1.9800 | |  | |
| 2.00 | 3 |  | |  | |  | | 2.1367 | |
| Significance |  | 1.000 | | 1.000 | | 1.000 | | 1.000 | |
| Display the group mean in the same subset of the table | | | | | | | | | |
| a. Harmonic mean sample size is used = 3.000 | | | | | | | | | |
| **h48** | | | | | | | | |  |
| Duncan^a^ | | | | | | | | |  |
| Concentrations | N | | A subset of alpha = 0.05 | | | | | |  |
|  |  |  | 1 | | 2 | | 3 | |  |
| 4.00 | 3 | | 1.3933 | |  | |  | |  |
| 3.00 | 3 | |  | | 1.5067 | |  | |  |
| 1.00 | 3 | |  | | 1.5133 | |  | |  |
| 2.00 | 3 | |  | |  | | 2.1800 | |  |
| Significance |  | | 1.000 | | .849 | | 1.000 | |  |
| **h72** | | | | | | | | | |
| Duncan^a^ | | | | | | | | | |
| Concentrations | N | | A subset of alpha = 0.05 | | | | | | |
|  |  |  | 1 | | 2 | | 3 | | 4 |
| 4.00 | 3 | | 1.0733 | |  | |  | |  |
| 3.00 | 3 | |  | | 1.2067 | |  | |  |
| 2.00 | 3 | |  | |  | | 1.3800 | |  |
| 1.00 | 3 | |  | |  | |  | | 1.4967 |
| Significance |  | | 1.000 | | 1.000 | | 1.000 | | 1.000 |
| **h96** | | | | | | | | |  |
| Duncan^a^ | | | | | | | | |  |
| Concentrations | N | | A subset of alpha = 0.05 | | | | | |  |
|  |  |  | 1 | | 2 | | 3 | |  |
| 4.00 | 3 | | .8333 | |  | |  | |  |
| 2.00 | 3 | |  | | 1.1833 | |  | |  |
| 3.00 | 3 | |  | | 1.1833 | |  | |  |
| 1.00 | 3 | |  | |  | | 1.4667 | |  |
| Significance |  | | 1.000 | | 1.000 | | 1.000 | |  |
| **h120** | | | | | | | | |  |
| Duncan^a^ | | | | | | | | |  |
| Concentrations | N | | A subset of alpha = 0.05 | | | | | |  |
|  |  |  | 1 | | 2 | | 3 | |  |
| 4.00 | 3 | | .5900 | |  | |  | |  |
| 3.00 | 3 | | .6667 | |  | |  | |  |
| 2.00 | 3 | |  | | .8233 | |  | |  |
| 1.00 | 3 | |  | |  | | 1.4467 | |  |
| Significance |  | | .094 | | 1.000 | | 1.000 | |  |
| Display the group mean in the same subset of the table | | | | | | | | |  |
| a. Harmonic mean sample size is used = 3.000 | | | | | | | | |  |

**2. ONEWAY CK C1 C2 C3 BY Time /STATISTICS DESCRIPTIVES /MISSING ANALYSIS /POSTHOC=DUNCAN ALPHA(0.05)**.

| STATISTICS DESCRIPTIVES | | | | | | | | | | | | | | |  |
| --- | --- | --- | --- | --- | --- | --- | --- | --- | --- | --- | --- | --- | --- | --- | --- |
| Time/Concentrations | | N | | Mean Value | | Standard deviation | | Standard Error | | 95% confidence interval for the mean | | | | Minimum | Maximum |
|  |  |  |  |  |  |  |  |  |  | Lower limit | | Upper limit | |  |  |
| CK | 24.00 | 3 | | 1.4833 | | .01528 | | .00882 | | 1.4454 | | 1.5213 | | 1.47 | 1.50 |
|  | 48.00 | 3 | | 1.5133 | | .05508 | | .03180 | | 1.3765 | | 1.6501 | | 1.46 | 1.57 |
|  | 72.00 | 3 | | 1.4967 | | .03786 | | .02186 | | 1.4026 | | 1.5907 | | 1.47 | 1.54 |
|  | 96.00 | 3 | | 1.4667 | | .03055 | | .01764 | | 1.3908 | | 1.5426 | | 1.44 | 1.50 |
|  | 120.00 | 3 | | 1.4467 | | .02517 | | .01453 | | 1.3842 | | 1.5092 | | 1.42 | 1.47 |
|  | Total | 15 | | 1.4813 | | .03833 | | .00990 | | 1.4601 | | 1.5026 | | 1.42 | 1.57 |
| C1 | 24.00 | 3 | | 2.1367 | | .01528 | | .00882 | | 2.0987 | | 2.1746 | | 2.12 | 2.15 |
|  | 48.00 | 3 | | 2.1800 | | .02000 | | .01155 | | 2.1303 | | 2.2297 | | 2.16 | 2.20 |
|  | 72.00 | 3 | | 1.3800 | | .03000 | | .01732 | | 1.3055 | | 1.4545 | | 1.35 | 1.41 |
|  | 96.00 | 3 | | 1.1833 | | .04509 | | .02603 | | 1.0713 | | 1.2953 | | 1.14 | 1.23 |
|  | 120.00 | 3 | | .8233 | | .07638 | | .04410 | | .6336 | | 1.0131 | | .74 | .89 |
|  | Total | 15 | | 1.5407 | | .55516 | | .14334 | | 1.2332 | | 1.8481 | | .74 | 2.20 |
| C2 | 24.00 | 3 | | 1.9800 | | .04583 | | .02646 | | 1.8662 | | 2.0938 | | 1.94 | 2.03 |
|  | 48.00 | 3 | | 1.5067 | | .03512 | | .02028 | | 1.4194 | | 1.5939 | | 1.47 | 1.54 |
|  | 72.00 | 3 | | 1.2067 | | .03512 | | .02028 | | 1.1194 | | 1.2939 | | 1.17 | 1.24 |
|  | 96.00 | 3 | | 1.1833 | | .03512 | | .02028 | | 1.0961 | | 1.2706 | | 1.15 | 1.22 |
|  | 120.00 | 3 | | .6667 | | .01528 | | .00882 | | .6287 | | .7046 | | .65 | .68 |
|  | Total | 15 | | 1.3087 | | .44694 | | .11540 | | 1.0612 | | 1.5562 | | .65 | 2.03 |
| C3 | 24.00 | 3 | | 1.8067 | | .04163 | | .02404 | | 1.7032 | | 1.9101 | | 1.76 | 1.84 |
|  | 48.00 | 3 | | 1.3933 | | .04726 | | .02728 | | 1.2759 | | 1.5107 | | 1.34 | 1.43 |
|  | 72.00 | 3 | | 1.0733 | | .05033 | | .02906 | | .9483 | | 1.1984 | | 1.02 | 1.12 |
|  | 96.00 | 3 | | .8333 | | .02082 | | .01202 | | .7816 | | .8850 | | .81 | .85 |
|  | 120.00 | 3 | | .5900 | | .05568 | | .03215 | | .4517 | | .7283 | | .54 | .65 |
|  | Total | 15 | | 1.1393 | | .44311 | | .11441 | | .8939 | | 1.3847 | | .54 | 1.84 |
| **One-way ANOVA** | | | | | | | | | | | | |  | | |
|  | | | Quadratic sum | | df | | Mean square | | F | | Significance | |  | | |
| CK | Intergroup | | .008 | | 4 | | .002 | | 1.604 | | .248 | |  | | |
|  | Intragroup | | .013 | | 10 | | .001 | |  | |  | |  | | |
|  | Total | | .021 | | 14 | |  | |  | |  | |  | | |
| C1 | Intergroup | | 4.296 | | 4 | | 1.074 | | 571.289 | | .000 | |  | | |
|  | Intragroup | | .019 | | 10 | | .002 | |  | |  | |  | | |
|  | Total | | 4.315 | | 14 | |  | |  | |  | |  | | |
| C2 | Intergroup | | 2.785 | | 4 | | .696 | | 576.901 | | .000 | |  | | |
|  | Intragroup | | .012 | | 10 | | .001 | |  | |  | |  | | |
|  | Total | | 2.797 | | 14 | |  | |  | |  | |  | | |
| C3 | Intergroup | | 2.729 | | 4 | | .682 | | 339.970 | | .000 | |  | | |
|  | Intragroup | | .020 | | 10 | | .002 | |  | |  | |  | | |
|  | Total | | 2.749 | | 14 | |  | |  | |  | |  | | |

**Similar subset after Duncan's repeat comparison**

| **CK** | | | | | |  | | |
| --- | --- | --- | --- | --- | --- | --- | --- | --- |
| Duncan^a^ | | | | | |  | | |
| Time (h) | N | | A subset of alpha = 0.05 | | |  | | |
|  |  |  | 1 | | |  | | |
| 120.00 | 3 | | 1.4467 | | |  | | |
| 96.00 | 3 | | 1.4667 | | |  | | |
| 24.00 | 3 | | 1.4833 | | |  | | |
| 72.00 | 3 | | 1.4967 | | |  | | |
| 48.00 | 3 | | 1.5133 | | |  | | |
| Significance |  | | .060 | | |  | | |
| Display the group mean in the same subset of the table | | | | | |  | | |
| a. Harmonic mean sample size is used = 3.000 | | | | | |  | | |
| **C1** | | | | | | | |  |
| Duncan^a^ | | | | | | | |  |
| Time (h) | N | A subset of alpha = 0.05 | | | | | |  |
|  |  | 1 | | 2 | 3 | | 4 |  |
| 120.00 | 3 | .8233 | |  |  | |  |  |
| 96.00 | 3 |  | | 1.1833 |  | |  |  |
| 72.00 | 3 |  | |  | 1.3800 | |  |  |
| 24.00 | 3 |  | |  |  | | 2.1367 |  |
| 48.00 | 3 |  | |  |  | | 2.1800 |  |
| Significance |  | 1.000 | | 1.000 | 1.000 | | .249 |  |
| **C2** | | | | | | | |  |
| Duncan^a^ | | | | | | | |  |
| Time (h) | N | A subset of alpha = 0.05 | | | | | |  |
|  |  | 1 | | 2 | 3 | | 4 |  |
| 120.00 | 3 | .6667 | |  |  | |  |  |
| 96.00 | 3 |  | | 1.1833 |  | |  |  |
| 72.00 | 3 |  | | 1.2067 |  | |  |  |
| 48.00 | 3 |  | |  | 1.5067 | |  |  |
| 24.00 | 3 |  | |  |  | | 1.9800 |  |
| Significance |  | 1.000 | | .430 | 1.000 | | 1.000 |  |
| **C3** | | | | | | | | |
| Duncan^a^ | | | | | | | | |
| Time (h) | N | A subset of alpha = 0.05 | | | | | | |
|  |  | 1 | | 2 | 3 | | 4 | 5 |
| 120.00 | 3 | .5900 | |  |  | |  |  |
| 96.00 | 3 |  | | .8333 |  | |  |  |
| 72.00 | 3 |  | |  | 1.0733 | |  |  |
| 48.00 | 3 |  | |  |  | | 1.3933 |  |
| 24.00 | 3 |  | |  |  | |  | 1.8067 |
| Significance |  | 1.000 | | 1.000 | 1.000 | | 1.000 | 1.000 |
| Display the group mean in the same subset of the table | | | | | | | | |
| a. Harmonic mean sample size is used = 3.000 | | | | | | | | |
