# Supplementary material for: Molluscicidal activity and physiological toxicity of quaternary benzo[c]phenanthridine alkaloids (QBAs) from Macleaya cordata fruits on Oncomelania hupensis
Source: PLoS Negl Trop Dis. 2019 Oct 11;13(10):e0007740. doi: 10.1371/journal.pntd.0007740 (PMC6808491; doi:10.1371/journal.pntd.0007740)
Supplement: S4 Fig — (DOC) [file pntd.0007740.s005.doc]

**Captions for figures**

**Fig. 4 Chromatogram of quaternary benzo[c]phenanthridine alkaloids in *Macleaya cordata* fruits determined by HPLC-MS.**

Note: The peak at 338 was an impurity peak in the HPLC-MS spectrometer.

**Fig. 5 Chromatogram of** **sanguinarine (SA) standard sample determined by HPLC-MS.**

**Fig. 6 Chromatogram of** **chelerythrine (CHE) standard sample determined by HPLC-MS.**


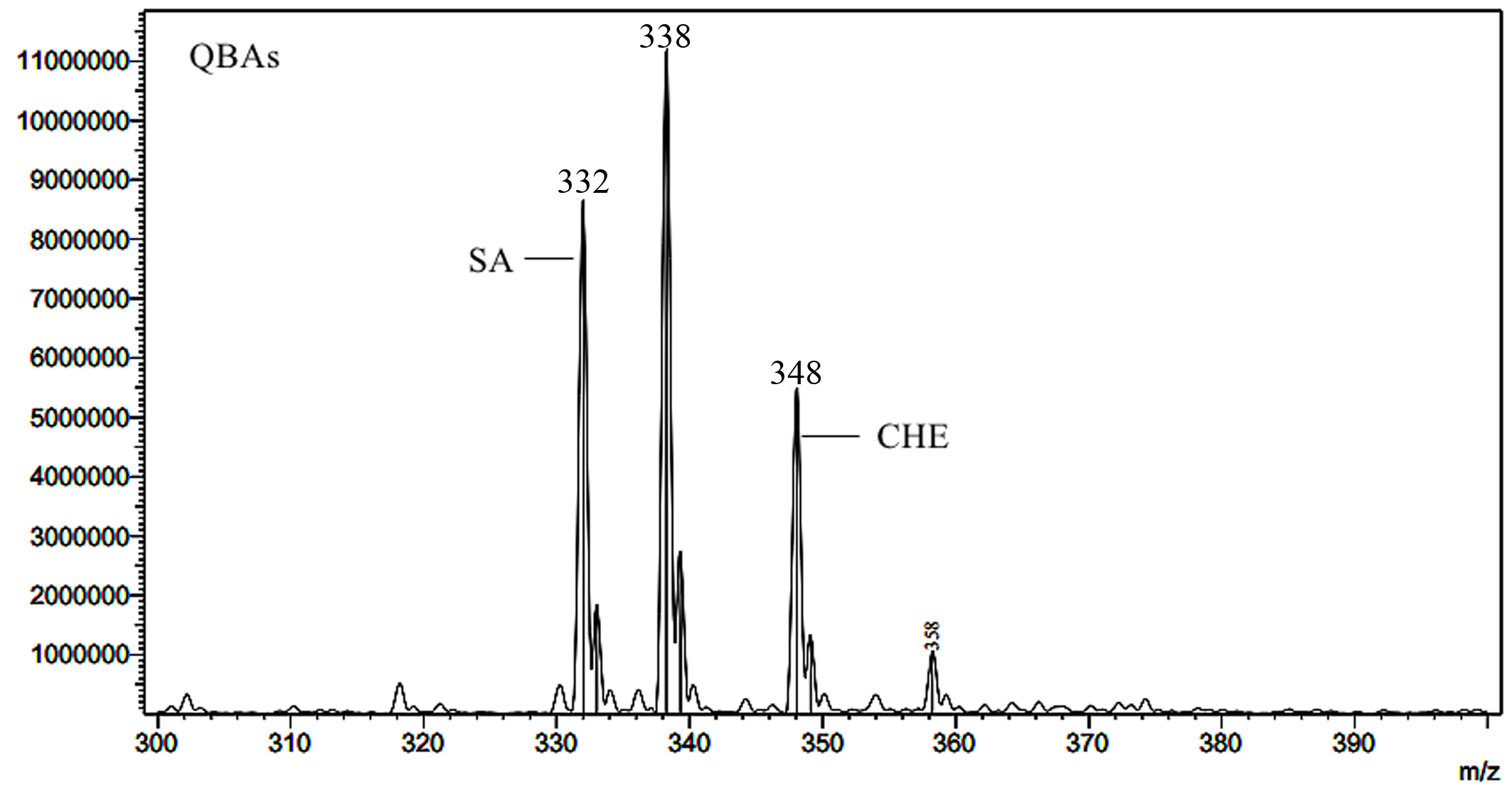


**Fig.4**

**
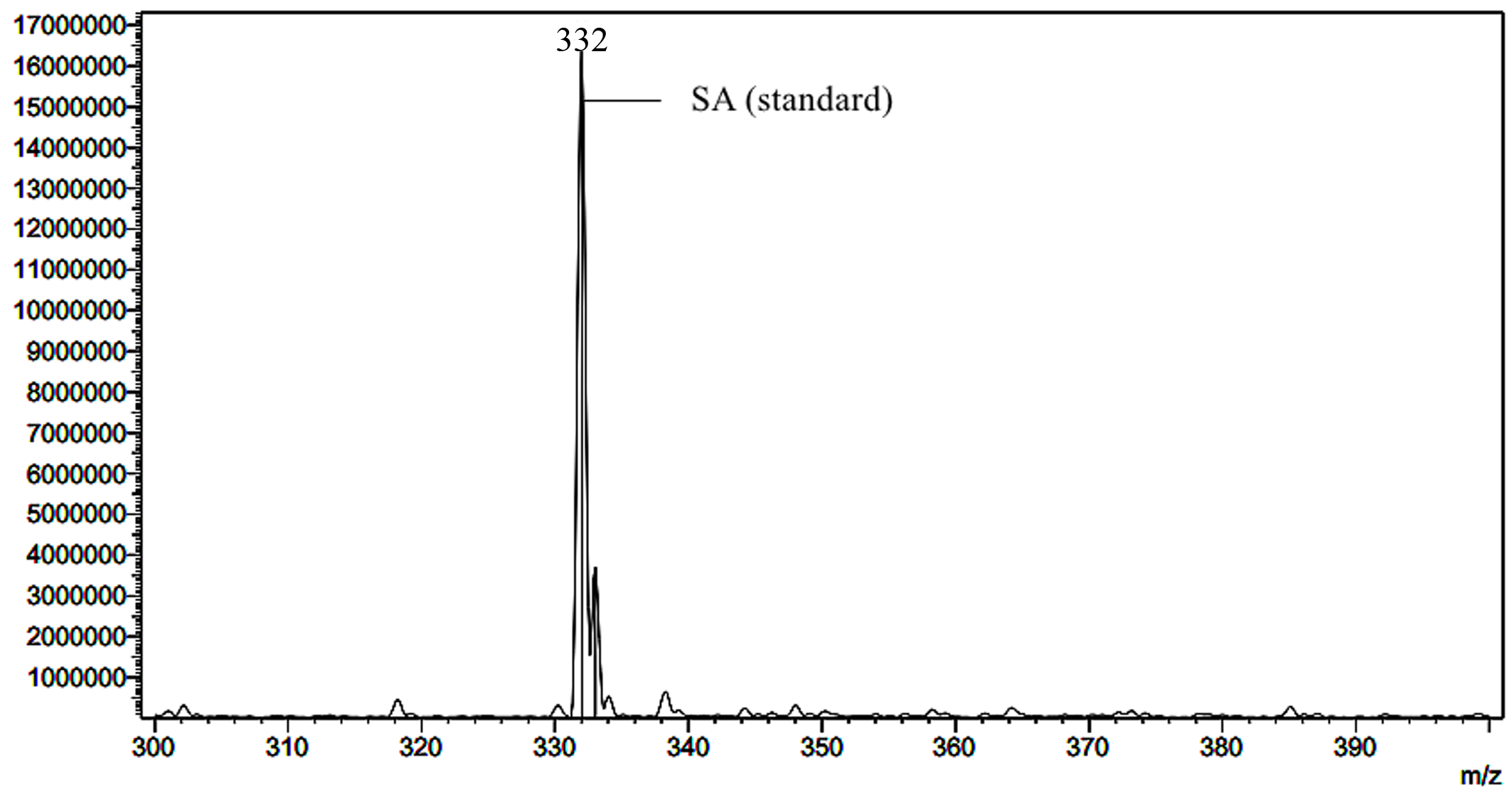
**

**Fig.5**

**
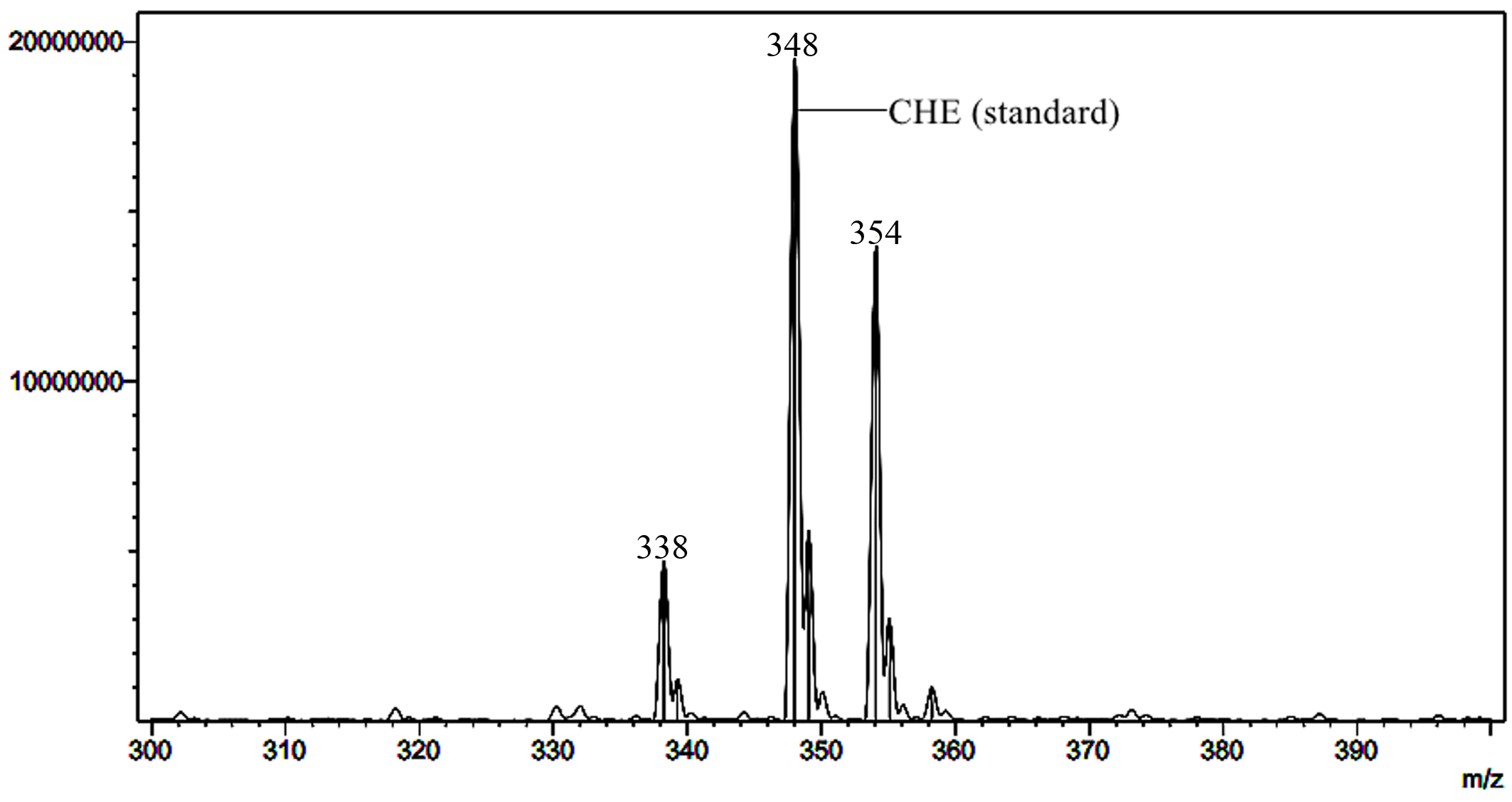
**

**Fig.6**
