# Supplementary material for: Molluscicidal activity and physiological toxicity of quaternary benzo[c]phenanthridine alkaloids (QBAs) from Macleaya cordata fruits on Oncomelania hupensis
Source: PLoS Negl Trop Dis. 2019 Oct 11;13(10):e0007740. doi: 10.1371/journal.pntd.0007740 (PMC6808491; doi:10.1371/journal.pntd.0007740)
Supplement: S5 Fig — (DOC) [file pntd.0007740.s006.doc]

**Captions for figures**

**Fig. 7 Mass spectrogram of quaternary benzo[c]phenanthridine alkaloids (BasePeak:332)**.

**Fig. 8 Mass spectrogram of quaternary benzo[c]phenanthridine alkaloids (BasePeak:348)**.

**Fig. 9 Mass spectrogram of sanguinarine (SA) standard sample**.

**Fig. 10 Mass spectrogram of chelerythrine (CHE) standard sample**.


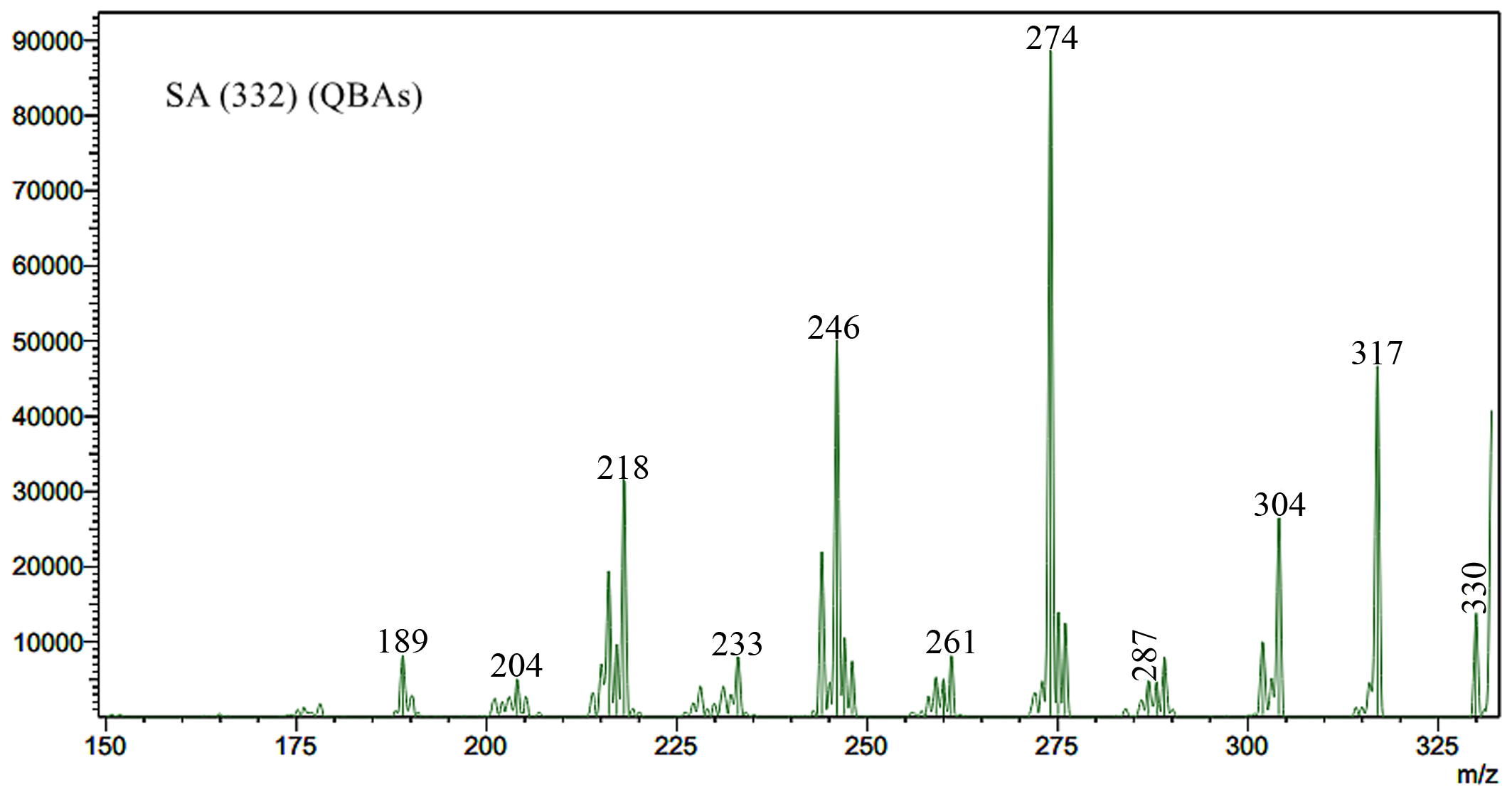


**FIG. 7**


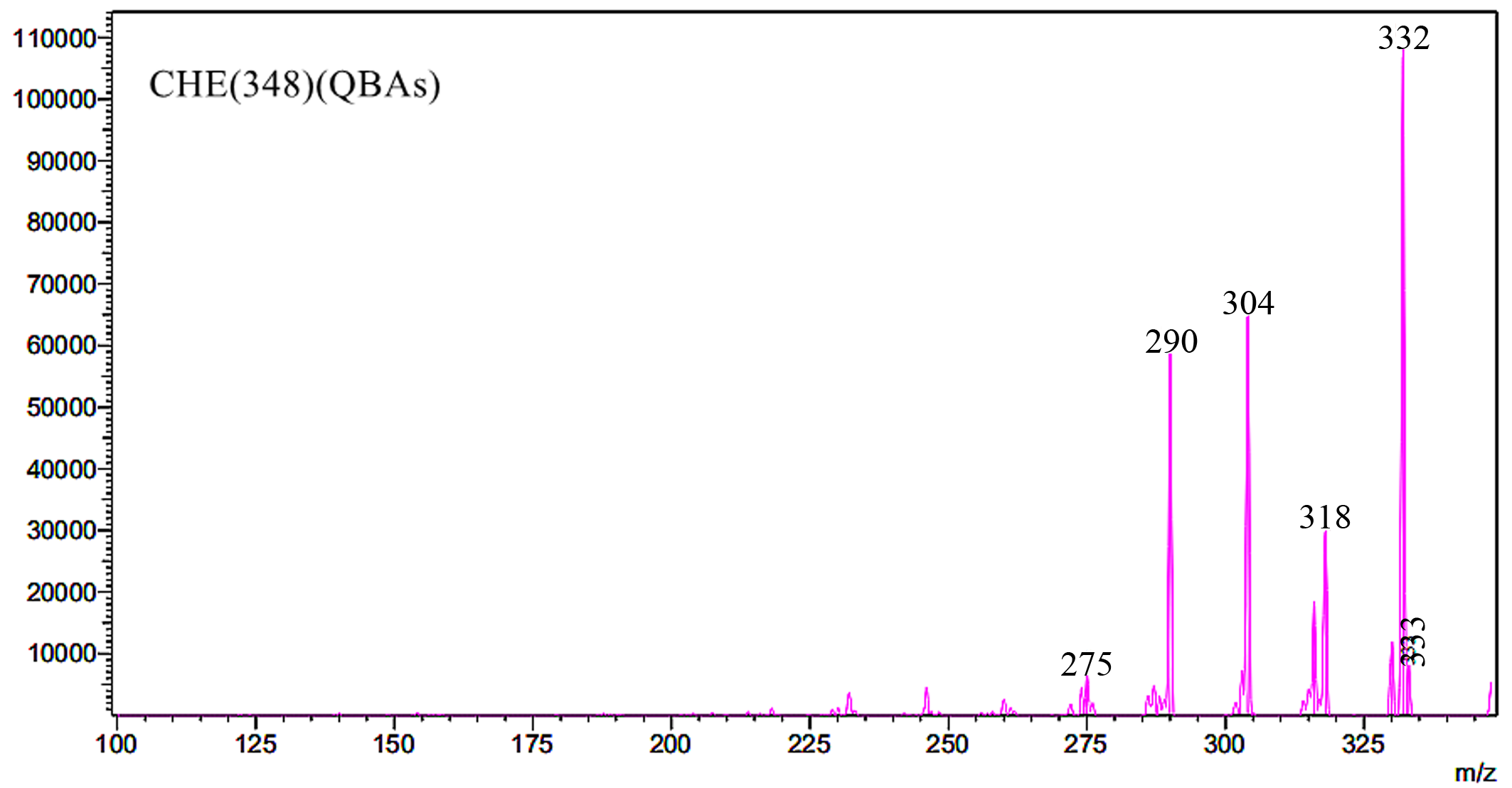


**FIG. 8**


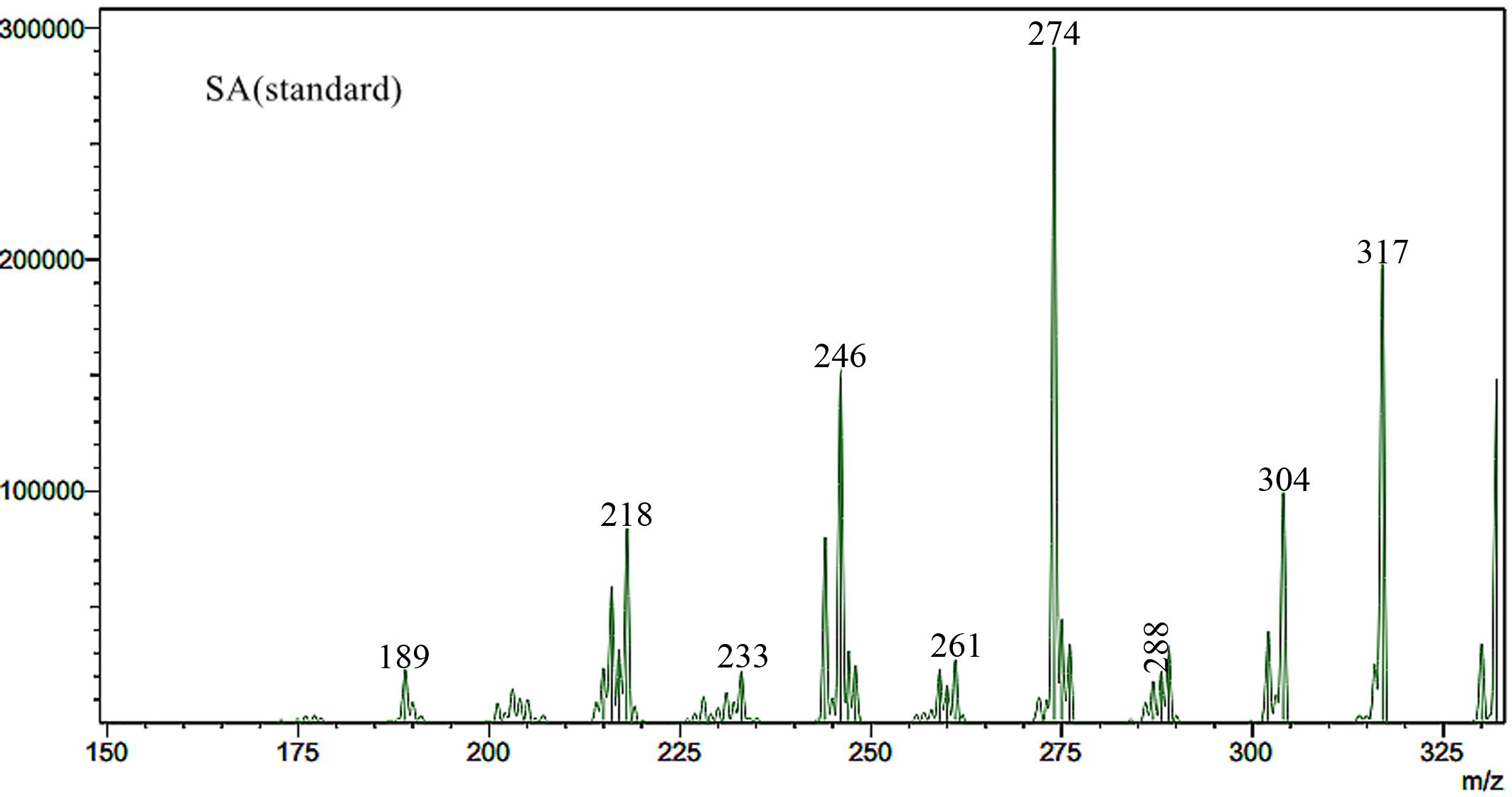


**FIG. 9**


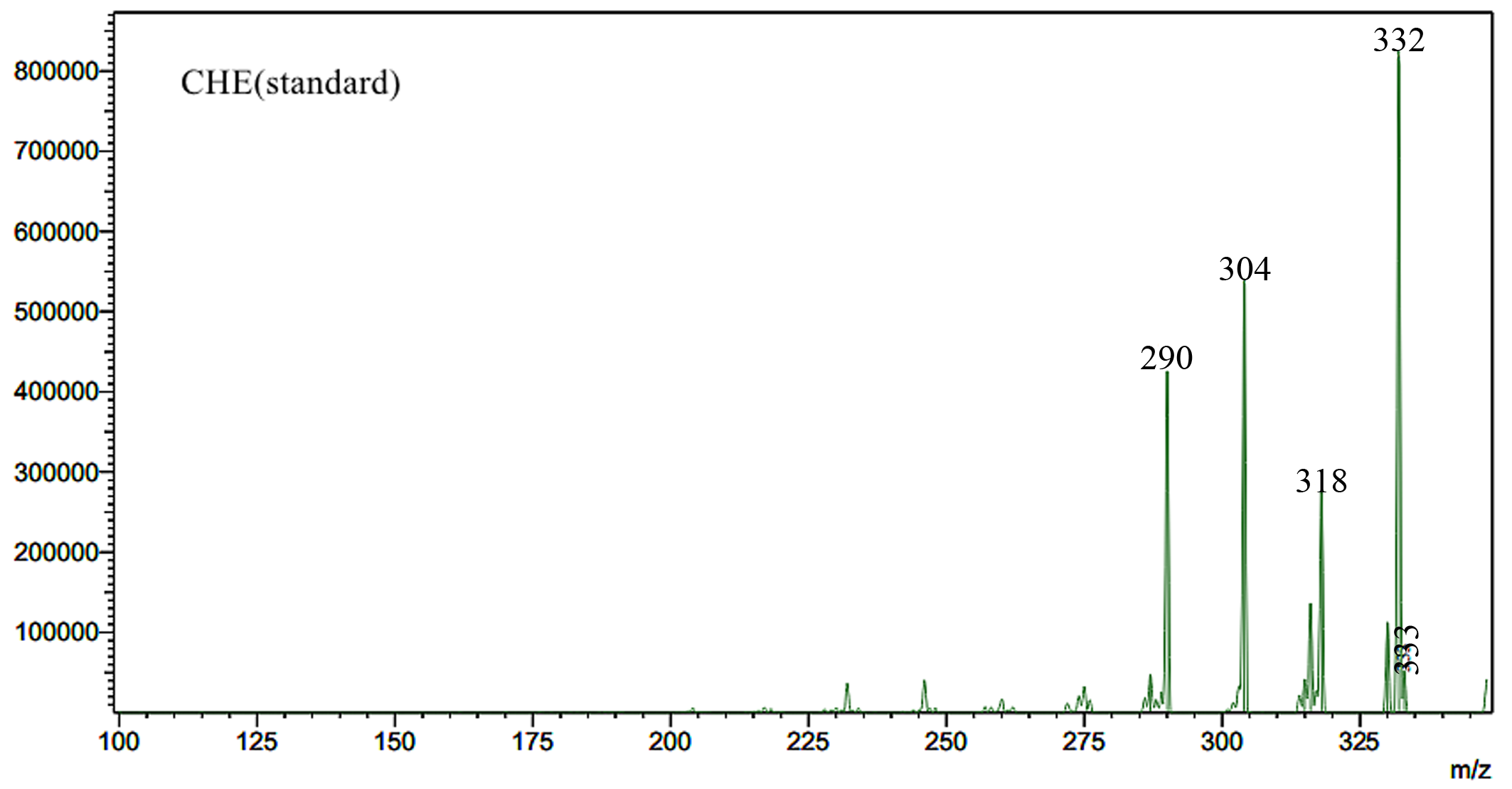


**Fig. 10**
